# Preconditioning donors with corticosteroids improves early lung graft immunity

**DOI:** 10.3389/fimmu.2025.1668591

**Published:** 2025-10-28

**Authors:** Isabelle Schwartz-Cornil, Florentina Pascale, Luc Jouneau, Maxime Huriet, Jérôme Estephan, Mickael Bourge, Christophe Richard, Valérie Gelin, Claudia Bevilacqua, Julie Rivière, Thien-Phong Vu Manh, Maxime Djebbour, Antoine Premachandra, Carla Gouin, Julien De Wolf, Chloé Mimbimi, Antoine Magnan, Antoine Roux, Stanislas Grassin-Delyle, Philippe Devillier, Delphyne Descamps, Nicolas Bertho, Sébastien Jacqmin, Morgan Le Guen, Edouard Sage, Matthieu Glorion

**Affiliations:** ^1^ Université Paris-Saclay, INRAE, UVSQ, VIM, Jouy-en-Josas, France; ^2^ Department of Thoracic Surgery and Lung Transplantation, Foch Hospital, Suresnes, France; ^3^ Cytometry/Electronic Microscopy/Light Microscopy Facility, Imagerie-Gif, Université Paris-Saclay, CEA, CNRS, Institute for Integrative Biology of the Cell (I2BC), Gif-sur-Yvette, France; ^4^ Université Paris-Saclay, UVSQ, INRAE, BREED, Jouy-en-Josas, France; ^5^ Université Paris-Saclay, AgroParisTech, INRAE, GABI, Jouy-en-Josas, France; ^6^ Université Paris-Saclay, INRAe, AgroParisTech, Micalis Institute, Jouy-en-Josas, France; ^7^ Aix Marseille University, CNRS, Inserm, CIML Centre d’Immunologie de Marseille-Luminy, Turing Center for Living Systems, Marseille, France; ^8^ Department of Pulmonology, Foch Hospital, Suresnes, France; ^9^ Université Paris-Saclay, UVSQ, INSERM, Infection et inflammation, U1173, Département de Biotechnologie de La Santé, Montigny-le-Bretonneux, France; ^10^ Exhalomics^®^ , Hôpital Foch, Suresnes, France; ^11^ Department of Pharmacology, Foch Hospital, Suresnes, France; ^12^ Oniris, INRAE, Bioepar, Nantes, France; ^13^ Department of Anesthesiology, Foch Hospital, Suresnes, France

**Keywords:** lung transplantation, innate immunity, macrophages, T cells, corticosteroids, translational research, preconditioning

## Abstract

**Background:**

Preclinical studies have recently revealed the critical role of innate immunity in determining lung transplantation outcomes. Although the International Society for Heart and Lung Transplantation recommends high-dose corticosteroid administration to donors, this practice is inconsistently applied worldwide. Investigating its impact on the donor lung’s innate immune response – an unexplored area - could provide valuable evidence to support adoption of donor preconditioning with corticosteroids, beyond their traditional administration to recipients.

**Method:**

We used a cross-circulatory pig platform that consists of a donor lung placed extracorporeally and connected to the circulation of a recipient pig whose leukocytes are fluorescently labeled.

**Results:**

Donor preconditioning - compared to recipient’s treatment alone - reduced the presence of CD3^pos^ T-cells in the graft from both the donor and recipient, and enhanced the anti-inflammatory profile of alveolar macrophages, at least during the first 10 hours of donor-recipient interaction. The alveolar macrophages isolated from corticosteroid-preconditioned pig lungs exhibited decreased gene expression of T-cell-attracting chemokines during the 10-hour reperfusion period, correlating with the reduced T-cell infiltration. Similarly, human lung macrophages showed lower expression of these T-cell-attracting chemokines and higher anti-inflammatory profiles with corticosteroid treatment.

**Conclusion:**

Our results show that the early immune status of lung grafts is improved by treating donors with corticosteroids through macrophage-targeted mechanisms. This finding provides an immunological rationale for expanding the implementation of donor preconditioning with corticosteroids.

## Introduction

Recent preclinical studies have underscored the pivotal role of the innate immune response – particularly involving monocyte/macrophage subsets - in determining the outcomes of lung transplantation (LT) (1–3). Several factors occurring during donor management at procurement can significantly affect this innate response, including duration of ischemia, ventilation-induced lung injury, endocrine and metabolic stress, infection and pharmacologic interventions (4). Among the latter, the administration of high-dose corticosteroids (CS, methylprednisolone) to donors has been supported by the Toronto team (5) and is recommended by the International Society of Heart and Lung Transplantation as part of donor management guidelines (6). This recommendation is based on a very limited number of studies that indicate benefits for lung oxygenation, increased success rates of procurements, and reduced lung water accumulation after LT (7, 8). However, the potential benefits of donor preconditioning with CS on the innate immune status of lung grafts are not yet known. While CS are systematically administered to the recipient during LT surgery, elucidating the immune effects of donor preconditioning could provide mechanistic support for current guidelines and promote more consistent implementation of steroid use in donors, particularly in regions such as Europe where these agents are not uniformly incorporated into standard donor management protocols.

CS are routinely prescribed for their potent anti-inflammatory and immunosuppressive properties, low cost and wide availability. They act through genomic and non-genomic pathways, with cell-specific effects on T cells, monocytes/macrophages, neutrophils, and epithelial cells (9). In order to investigate the early immune effects of lung donor preconditioning with CS, we employed a cross-circulation platform that involves connecting an extracorporeal donor pig lung to the blood circulation of a recipient pig harboring fluorescently labeled blood cells (10). This platform provides a simple, reproducible, controllable and ethically sound alternative to full transplantation, with the advantage of enabling longitudinal and comparative analysis of donor and recipient immune responses during the early phases of alloimmune interactions in pigs, a species recognized as the primary translational model in LT research (11). We found that donor preconditioning improved the immunological status of the graft, by modulating the gene expression in alveolar macrophages (AMs) and reducing the graft content in T cells, providing an immunological rationale for donor preconditioning with CS.

## Material and methods

### Animals and their conditioning

**Figure 1:**
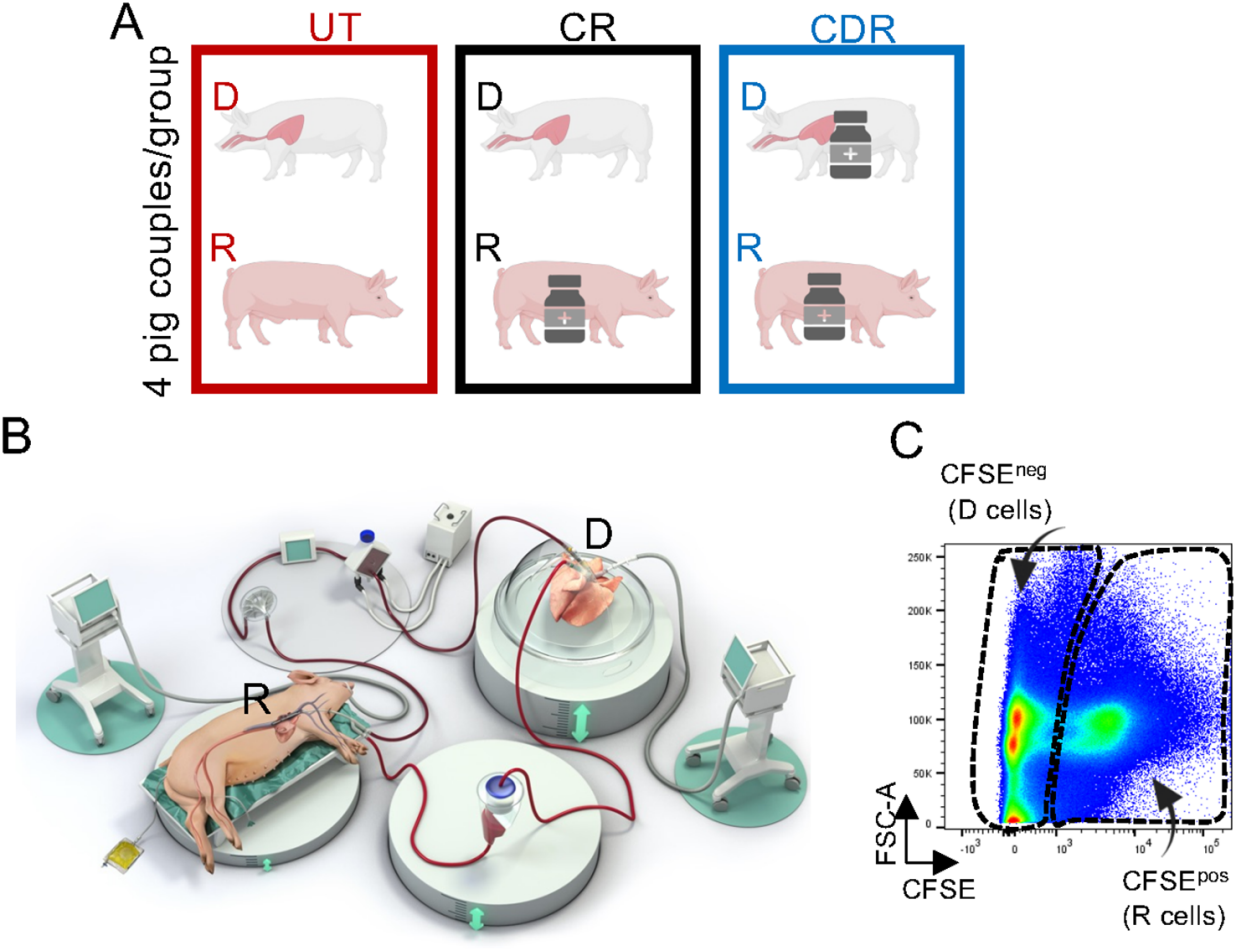
Experimental set up. **(A)** Pigs (donors (D) and recipients (R)) were split in 3 groups: UT includes donors and recipients that received no treatment, CR includes recipients that received 20 mg/kg methylprednisolone 60 min before cross-circulation, and CDR includes donors that received 20 mg/kg methylprednisolone 12 h before lung procurement and recipients that received 20 mg/kg methylprednisolone 60 min before cross-circulation. **(B)** The cross-circulation’s experimental set up has been previously described in (10). CFSE (25 mg) was injected 30 min before cross-circulation, and it was shown to label the blood cell compartment (10). Blood and lung tissue biopsies were collected at different time points. **(C)** Flow cytometry profile of isolated cells from a lung biopsy 6 h after the onset of cross-circulation; the gates show the CFSE^pos^ cells (recipient (R)) and CFSE^neg^ cells (donor (D)).

Large White pigs (3–4 months-old, 24 in total) included matched pairs of male donors and female recipients originating from unrelated parental origins. They were split into three experimental groups, four pig couples per group (**Figure 1**): untreated donors and recipients (UT), CS-treated recipients only (CR, recipients that received 20 mg/kg methylprednisolone IV (Mylan, Canonsburg, USA) 60 min before cross-circulation) and CS-treated donors and recipients (CDR, donors that received 20 mg/kg methylprednisolone IV 12 h before lung procurement and recipients that received 20 mg/kg methylprednisolone 60 min before cross-circulation).

### Cross-circulation procedure

The overall cross-circulation procedure has been detailed in (10) based on a model previously established by the Columbia University team (12). The lung harvest from donor pigs (*n* = 12) was performed as described (10) in the Animal Surgery and Medical Imaging Platform (CIMA-MIMA2-BREED-INRAE, Jouy en Josas, France). Recipient pigs (*n* = 12) were anesthetized as described (10) and Septotryl^®^ (0.08 ml/kg) (Vetoquinol) was injected IM prior to catheterization. Heparin was administered as an initial 25,000 U bolus, and maintained by continuous infusion (100 U/Kg/h). The superior vena cava was cannulated with a 20 F double lumen cannula as described (10). Carboxyfluorescein succinimidyl ester (CFSE, 25 mg, Sigma-Aldrich, Saint-Louis, Mississippi, USA) was administered IV in the perfusing pig, diluted in 4 ml DMSO + 40 µl heparin, 30 minutes before the initiation of cross-circulation. Donor lungs were placed in dorsal position on an XVIVO^®^ chamber (XVIVO Perfusion) and the trachea was cannulated with a 7.5 mm diameter cuffed endotracheal tube (Mallinckrodt, Staines-upon-Thames, UK) and connected to a respirator. The vascular tubing was spliced to connect the recipient pig to the dedicated circuit, marking the start of cross-circulation. Sedation was kept for 10 hours by permanent administration of 2 mg/kg/h propofol (Proposure^®^, Axience, Pantin, France) with 0.6% isofluorane and analgesia was achieved by IV administration of 0.2 mg/kg nalbuphine every 3 hours.

### Extracorporeal hemodynamic and lung function monitoring

We collected blood samples from the pulmonary artery and vein (PA and PV) cannula hourly to perform the hemo-gas analyses using an Istat^®^ kit. The static compliance (Tidal Volume/(plate pressure – positive end expiratory pressure)), ΔPCO2 (arterial PCO2 – venous PCO2) and ΔPO2/FIO2 (venous PO2 – arterial PO2/FIO2) were measured once per hour. The transpulmonary pressure (PA - PV) and the pulmonary vascular resistance were calculated (PA pressure – left atrial pressure) x 80/flow rate.

### Blood sample collection

We collected blood samples directly from the extracorporeal circuit. Blood counts and biochemical profiling from plasma were performed on a MS4.5 analyzer and a M-Scan II analyzer (Melet Schloesing Laboratoires, Cergy-Pontoise, France).

### Lung biopsies from pigs

Lung biopsies for cell dissociation and RNA extraction were sampled at T0H, T6H and T10H of cross-circulation using a surgical stapler (Endo GIA™ universal stapling system, Medtronic, Minneapolis, USA). For cell dissociation, 2 g of tissue were immediately immerged in cold hypothermic preservation media (HypoThermosol^®^ FRS, Stemcell Technologies Inc, Vancouver, Canada) and for RNA, about 50 mg of tissue were stored in RNAlater (Thermo Fisher Scientific, Waltham, MA, USA) at -20 °C. For histology, biopsies were fixed in cold phosphate-buffered 4% paraformaldehyde for 24 hours and were subsequently paraffin-embedded.

### Lung biopsies from human patients

Lung tissue samples were obtained from eight patients undergoing surgical resection for lung carcinoma at the Foch Hospital in Suresnes (France) (**Additional File 1**). The lung tissue parts located away from the tumor were dissected and used for cell isolation.

### Lung cell isolation

Lung tissue was finely chopped and incubated at 37 °C for 45 minutes under rotation in RPMI 1640 supplemented with 100 IU/ml penicillin,100 mg/ml streptomycin, and 10% inactivated fetal calf serum (FCS) (Invitrogen, Paisley, UK), containing 3 mg/ml collagenase D, 0.25 mg/ml Dnase I (Sigma-Aldrich) and 0.7 mg/ml dispase II (Gibco^®^, ThermoFisher Scientific, St Aubin, France). The cell preparation was subsequently filtered on a nylon mesh (1 mm diameter) followed by successive passages through cell strainers (500 µm, 100 µm, 40 µm). Erythrocytes were eliminated with an erythrocyte lysis buffer (10 mM NaHCO3, 155 mM NH4Cl, and 10 mM EDTA). The cells were washed in PBS, counted and 10^8^ cells were processed to cell surface staining. For subsequent pig cell sorting, the remaining cells were frozen in 10% DMSO + 90% FCS using a Mister Frosty freezing container (Nalgene, Rochester, NY, USA) and finally kept in liquid N2.

### Purification of macrophages from isolated human lung cells cultured with and without corticosteroids

The isolated human lung cells were suspended at a concentration of 2 x 10^6^ cells/ml in RPMI containing 10% fetal calf serum, 100 U/ml penicillin and 100 µg/ml streptomycin, with or without 20 µg/ml methylprednisolone, for 12 or 42 hours (Sigma-Aldrich Merck Group, MA, USA). After culture, the lung cells were harvested using accutase treatment (Sigma-Aldrich Merck Group) and spun at 600 g. The cells (30 to 40x10^6^) were then incubated with an anti-CD172A mAb (DH59B clone, 5 µg/ml) for 30 minutes at 4 °C and the positively labeled cells were magnetically sorted using anti-mouse IgG coated beads and LS columns (Myltenyi, Bergisch-Gladbach, Germany) following the manufacturer’s recommendations. The purity of the CD172A^pos^ cells was > 92% estimated by flow cytometry control.

### Pig cell surface staining and analysis by flow cytometry

We performed the cell surface staining in RPMI supplemented with 10 mM Hepes, 5% horse and swine serum respectively (Gibco, Life Technologies Europe, Bleiswijk, Netherlands). We used swine-specific primary antibodies (Abs) and conjugated secondary Abs that are listed in **Additional File 2**. We used isotype controls (mouse IgG1, IgG2b, and IgG2a) at the same concentration as the tested mAbs, based on the fluorescence minus one method (13). In some cases, a directly PE-conjugated IgG1 mAb (anti-CD163-PE and anti-CD3-PE) was used as a third step, and an excess of mouse IgG1 (50 μg/ml) was added to saturate the first IgG1 step. A DAPI staining (Sigma-Aldrich) was used to exclude dead cells or to assess cell death among CD3^pos^ T cells. Results were acquired on BD LSR Fortessa™ Cell Analyzer (BD-Biosciences). The FACS data were analyzed with the FlowJo software (version 10.7.1; Tree Star, Ashland, OR, USA). The gating strategy is shown in **Additional File 3** that mainly originates from our recent paper (10).

### Cell sorting of pig alveolar macrophages, CD14^pos^ and CD16 ^pos^ monocytic cells

Isolated pig lung cells were thawed from frozen stocks and reacted with anti-CD14, CD16 and CD172A mAbs followed by conjugated goat anti-mouse isotype-specific Abs (**Additional File 2**). The anti-IgG1 conjugate was saturated by an excess of mouse IgG1 (50 μg/ml) and cells were labeled with anti-CD163-PE (IgG1). The cell subsets were sorted (“purity” mode) by flow cytometry on the Imagerie-Gif Cytometry facility (I2BC, Gif sur Yvette, France) using the MoFlo ASTRIOS sorter (Beckman-Coulter, Paris, France) and the Summit 6.3 software. Alveolar macrophages (AMs) were sorted as CFSE^neg^SSC^hi^CD163^hi^/CD172A^hi^ from total live lung cells. The MoCs were sorted as CFSE^pos^SSC^lo^CD172A^hi^CD14^pos^CD16^lo^ (named CD14^pos^) and CFSE^pos^SSC^lo^CD172A^hi^CD14^lo^CD16^hi^ cells (named CD16^pos^).

### RNA extraction, reverse-transcription and RT-qPCR

RNA from flow cytometry-sorted pig CD14^pos^ MoCs, CD16^pos^ MoCs and AMs as well as from immunomagnetically-sorted human CD172A^pos^ lung macrophages were extracted using the Arcturus PicoPure™ RNA kit (ThermoFisher Scientific). Calibrator RNA samples from pig and human lung cells were prepared in parallel. In addition, whole lung tissue biopsies collected in RNAlater were placed in Trizol, homogenized with 1.4 mm ceramic beads in a Precellys 24 bead grinder homogenizer (Bertin Technologies, St Quentin en Yvelines, France), and purified using the NucleoSpin RNA kit that includes a DNAse digestion step (Macherey-Nagel, Düren, Germany). RNA was quantified by Qubit™ RNA high sensitivity kit (Invitrogen™, Fisher Scientific SAS, Illkirch, France). In the case of sorted cells, RNA (8–100 ng) was reverse transcribed using random primers (5 µM) and oligo dT primers (2.5 µM) and the Multiscribe reverse transcriptase (Applied Biosystem, ThermoFisher Scientific), using equal starting quantities of RNA from test and calibrator RNA. Quantitative real-time PCR was carried out with 300 nM primers in a final reaction volume of 25 µl of 1 X SYBR Green PCR Master Mix (Applied Biosystem, ThermoFisher Scientific). In the case of lung tissue, RNA (400 ng) was reverse-transcribed using random primers (5 µM) and oligo dT (2.5 µM) primers and the Takara Prime Script RT reagent kit (TaKaRa, Kyoto, Japan). Quantitative real-time PCR was carried out with 300 nM primers in a final reaction volume of 25 µl of Q-PCR TB Green Premix ExTaq (TaKaRa). PCR cycling conditions were 95 °C for 30 seconds, linked to 40 cycles of 95 °C for 5 seconds and 60 °C for 30 seconds. The primers were designed using the primer express software (v2.0) or were bought from Sigma-Aldrich and are reported in **Additional File 4**. Real-time qPCR data were collected by the Bio-Rad CFX Maestro system (Bio-Rad Laboratories Inc, Marne-la-Coquette, France) and expression of the different genes was calculated relatively to RPS24 expression in pig and to RPS18 in human. All PCR data were normalized to the internal calibrator (arbitrary units) and the values were calculated by the 2^−ΔΔ^CT method.

### Immunohistochemistry

Formalin-fixed paraffin-embedded lung tissues at T0H from the UT and CDR groups were cut every 50 µm to produce three 5 µm tissue slices per sample. After antigen unmasking (100 °C, 20 minutes), the tissue sections were incubated with 30% H2O2, followed by Avidin/biotine (OriGene, Rockville, USA), saturated with 10% goat serum (Vector Laboratories, Newark, USA), reacted with 2.5 µg/ml anti-pig CD3 (clone PPT3, Clinisciences) followed by 1:400 dilution of biotinylated Goat anti-mouse IgG (Vector Laboratories) in Bond™ Primary Antibody Diluent (Leica, Wetzlar, Germany), revealed by the VECTASTAIN ELITE ABC HRPO and DAB substrate kits (Vector Laboratories) and counterstained with Hematoxylin (Diapath, Martinengo, Italy). The slides were imaged with a slide scanner (Pannoramic SCAN II, v3.0.2, 3DHistech, Medipixel Ltd, Budapest, Hungary). Ten randomly selected high-power fields per slide (0.1 mm^2^ area) were observed from three slides per sample and the mean number of CD3^pos^ T cells per pig was calculated.

### Human CXCL10 detection

The human lung cell supernatants were thawed, spun at 10000 g and assayed using the Human CXCL10/IP-10 DuoSet ELISA (R&D Systems, MI, USA).

### scRNA-seq and processing of sequencing data

Freshly isolated pig lung cells from an untreated pig (2 x 10^7^ cells) were incubated with anti-MHC class II (MSA clone, 2 µg/ml) for 30 minutes, washed, and reacted for 15 minutes with 20 µl goat anti-mouse IgG-conjugated immunobeads (Myltenyi, Bergisch-Gladbach, Germany). After filtration on a 40 µm cell strainer, MHC class II^pos^ cells were enriched with a MS Myltenyi separation column (ref 130-042-201), following the manufacturer’s recommendations. Cells were counted using a counting chamber and checked for viability using trypan blue and showed over 90% viability. A cell sample (2 x 10^4^) was loaded onto the 10x Chromium to produce a sequencing library, which was processed according to methods provided by 10x Genomics 3’ end (v3 Chemistry, 3’ end). Cell cDNA was sequenced using the Truseq Illumina Stranded protocol and the Illumina NextSeq 500 sequencing machine (> 3x10^8^ reads/sample). The reads were aligned with Cell Ranger v3.0.1 on the pig genome assembly version 11.1 using the Ensembl release 99 of gene annotations. The sequencing results were pre-processed and normalized using Seurat v4.3.0. Cells expressing less than 500 genes were removed. A total of 11029 cells were used to generate an UMAP with clustering (dimensions of reduction used = 11, k.param nearest neighbors = 20, resolution = 0.4). The top differentially expressed genes in each cluster were extracted (log 2-fold change > 0.6) using the FindAllMarker function of Seurat (**Additional File 5**), after removal of the ribosomal genes. The most highly expressed genes were then used to determine the cell type of each cluster, based on the expression of recognized hallmark genes for human lung (14–16).

### Study approval and ethics

The surgery was conducted at the Medical Imaging in Animal platform (accreditation B78-322-2) and the animals were hosted at the Animal Genetics and Integrative Biology unit at INRAE-Jouy (accreditation C78-719). The animal experiments were conducted in accordance with the EU guidelines and the French regulations (DIRECTIVE 2010/63/EU, 2010; Code rural, 2018; Décret n°2013-118, 2013). The experiments were approved by the COMETHEA ethic committee under the APAFIS number authorization 25174–2020011414322379 and were authorized by the French “ministère de l’enseignement supérieur et de la recherche”. The human part of the study was declared as “dossier de Conservation et de préparation à des fins scientifiques D’Eléments du Corps Humain”: (CODECOH) DC N° DC-2020-3981 (1). Experiments on human tissues were approved by the regional investigational review board (Comité de Protection des Personnes Île de France VIII, Boulogne-Billancourt, France). In line with the French legislation on clinical research and as approved by the investigational review board, all the patients gave their informed consent for the use of resected lung tissue for research.

### Statistics

For the physiological and biological parameters, data were Log10-transformed and analyzed with R. Statistical comparisons were performed across time points and between control and treated groups using repeated-measures ANOVA, when the assumptions for parametric testing were met. A Shapiro test was used to evaluate the normality of the data distribution in each group and timing. When normality was confirmed, a t-test was used following equal variance evaluation. If normality was not met, the non-parametric Wilcoxon signed-rank test was applied. Paired tests were performed for comparison between timing and unpaired tests were performed for comparison between groups. The p-values were systematically adjusted for multiple comparisons using the Bonferroni correction. The statistics of the genomic data are reported in the dedicated paragraph. Mean ± standard deviations were calculated.

## Results

### Preconditioning the donor with CS reduces the amount of donor and recipient CD3^pos^ T cells in lung grafts

Using a cross-circulation pig model, we compared the early cellular responses in lung grafts between three groups (**Figure 1**): untreated donors and recipients (UT), CS-treated recipients only (CR) and CS-treated donors and recipients (CDR). The CR group represents the widely adopted standard of care. The respiratory functions and the blood biological parameters were similar and globally stable in the three groups during the 10-hour experiments, indicating that the CS treatments did not impact the lung physiology (**Additional Files 6**, **7**). The representation of immune cell subsets was analyzed in the three groups, considering cells from the recipient (CFSE^pos^) and from the donor (CFSE^neg^) (**Additional File 3**, **Figure 1**). The pig lung immune subsets encompass CD3^pos^, CD4^pos^ and CD8^pos^ T cells, NK cells, B cells, CD172A^hi^ monocytic cells (MoCs) including the CD14^pos^ and CD16^pos^ monocyte subsets (classical and non-classical/intermediate, respectively) (11), and CFSE^neg^ CD163^hi^ lung macrophages that mainly correspond to alveolar macrophages (AMs) (11, 17).

**Figure 2:**
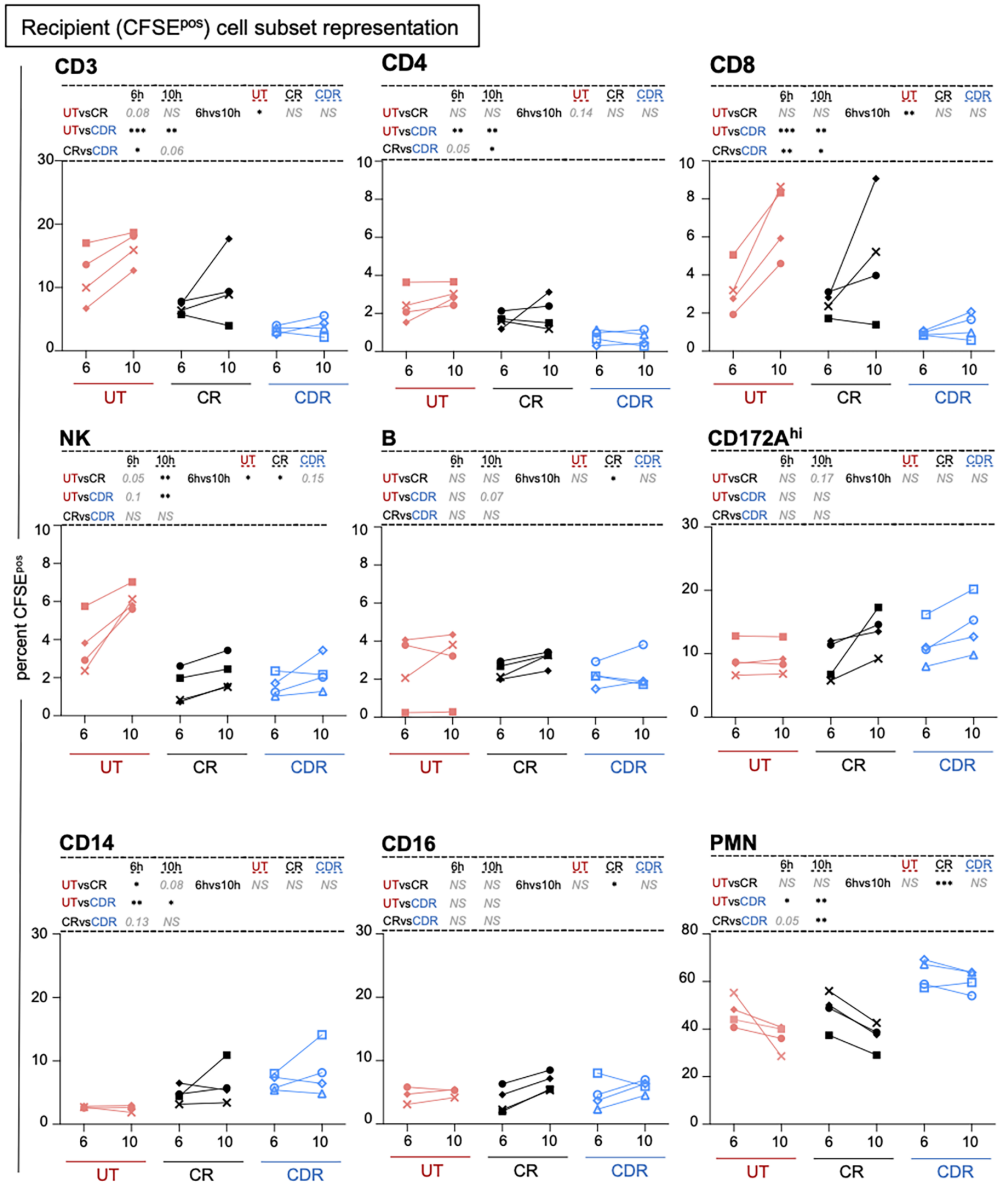
Preconditioning the donor with CS modifies the composition of the cell subsets recruited from the recipient in the lung graft. Cells were gated as presented in the workflow (**Additional File 3**) and the percent among CFSE^pos^ cells is reported. Each pig is labeled with a unique colored symbol throughout the paper (UT no treatment in red, CR corticosteroids to recipient only in black, CDR corticosteroids to donor and recipient in blue). When the log-transformed data followed a normal distribution, a paired t-test was performed to compare the data between timing and an unpaired t-test was performed to compare the data between groups; alternatively, a Wilcoxon test was performed. The p-values were corrected for multiple testing, * < 0.05, ** < 0.01, *** < 0.001, NS stands for non-significant, p-values > 0.05 and < 0.2 are indicated. The mean and sd values are reported in **Additional File 9**.

Notably, the global recruitment of the recipient’s cells (CFSE^pos^) did not differ between the three groups (21.6 to 30.7% CFSE^pos^ cells among live cells at T10H, **Additional Files 8**, **9**). As reported in our previous study, the administration of CS to the recipient only had a minor influence on the recruited cell subsets’ composition (11). Interestingly, preconditioning the donor strongly reduced the amount of CD3^pos^ T cells among the recipient cells recruited in the graft (3.9 ± 1.4 in CDR vs 16.3 ± 2.7 in UT at T10H, adj-p < 0.01 with no significant difference between CR and UT), affecting both the CD4^pos^ and CD8^pos^ T cell compartment (**Figure 2**, **Additional File 9**). The B cell and CD16^pos^ MoC representation was not significantly changed by the treatments. Similarly in the CR and CDR compared to in the UT group, the NK cell representation decreased at T10H and the CD14^pos^ MoC representation increased at T6H. The PMN representation appeared higher in the CDR than in the CR group (**Figure 2**, **Additional File 9**).

**Figure 3:**
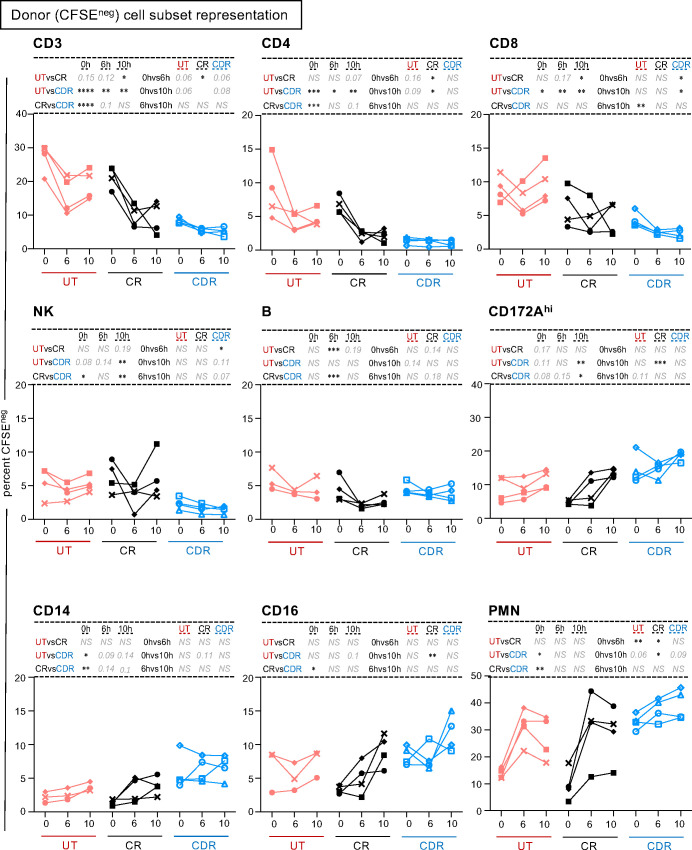
Preconditioning the donor with CS modifies the representation of the donor cell subsets in the lung graft. Cells were gated as presented in the workflow (**Additional File 3**) and the percent among CFSE^neg^ cells is reported. Each pig is labeled with a unique colored symbol throughout the paper (UT no treatment in red, CR corticosteroids to recipient only in black, CDR corticosteroids to donor and recipient in blue). When the data followed a normal distribution, a paired t-test was performed to compare the data between timing and an unpaired t-test was performed to compare the data between groups, alternatively a Wilcoxon test was performed. The p-values were corrected for multiple testing, * < 0.05, ** < 0.01, *** < 0.001, NS stands for non-significant, p-values > 0.05 and < 0.2 are indicated. The mean and sd values are reported in **Additional File 9**.

**Figure 4:**
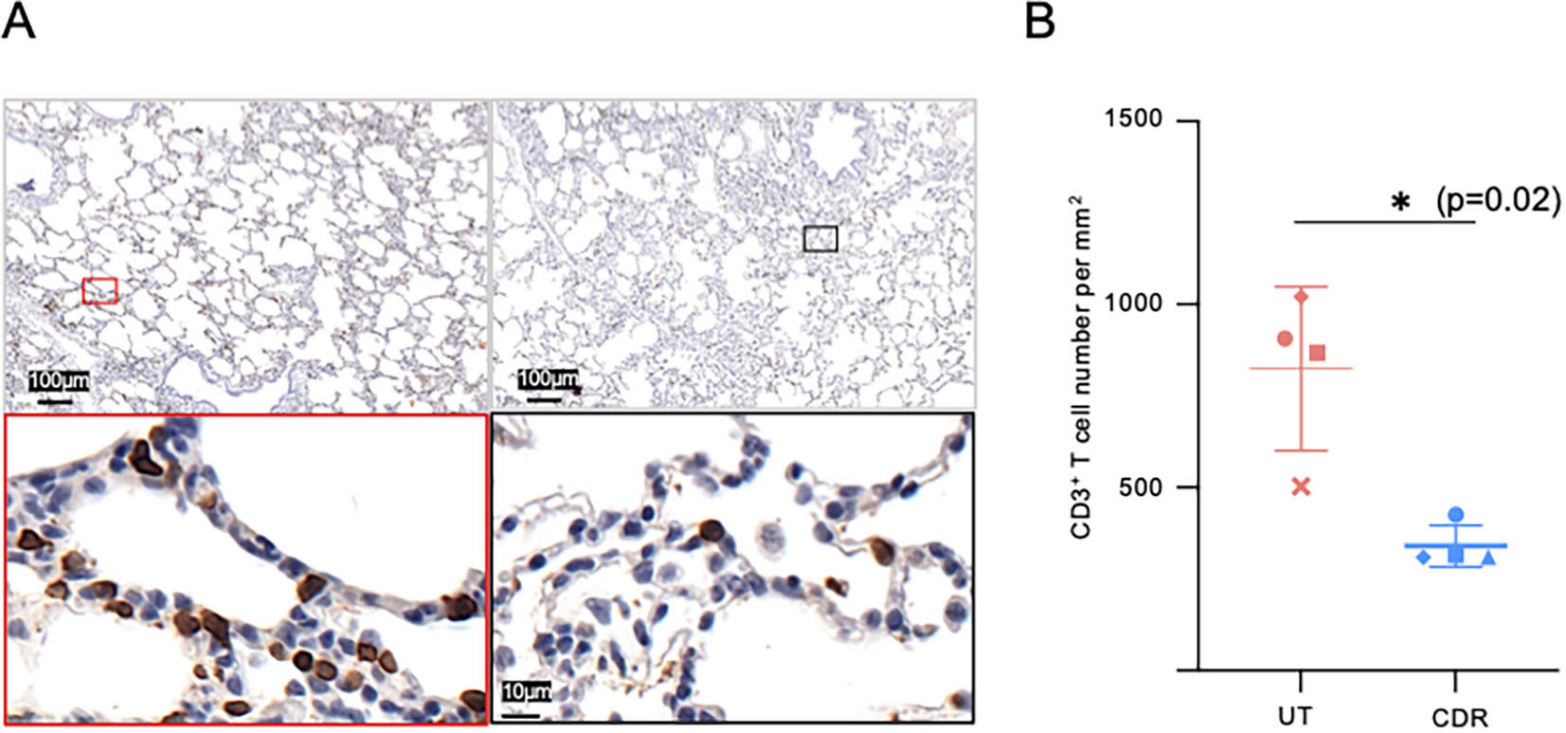
Reduction of donor CD3^pos^ T cells in lungs by the corticosteroid treatment. **(A)** Representative photograph of immunohistochemical CD3 staining of untreated (UT group, left) and corticosteroid-treated (right, CDR group) donor pig lungs. The lung samples were obtained upon pig donor death, 12 h post-methylprednisolone injection (CDR). **(B)** The mean number of CD3+ T cells per mm^2^ in 4 pigs per group was calculated from ten randomly-selected high power field (HPF, 0.1 mm^2^ area) per slide, 3 slides per sample. As the data distribution did not pass the normality test, a two tailed Wilcoxson test was performed. (*, p-value < 0.05).

Regarding the donor cells (CFSE^neg^), we observed that preconditioning the donor modified the donor cell composition in the graft at T0H, resulting in a decreased representation of CD3^pos^ T cells and NK subsets and a higher representation of CD14^pos^ MoC and PMN cells (**Figure 3**, **Additional File 9**). The decrease in CD3^pos^ T cells by CS in the CDR group was confirmed by immunohistochemistry (p < 0.05, **Figure 4**). The cross-circulation alone modified the donor cell subsets’ representation over time in the three groups, with a tendency toward reduction of CD3^pos^ T cells and increase in PMN and MoC representation (**Figure 3**, **Additional File 9**). However, the CD3^pos^ cell percent among donor cells reached the lowest values in the CDR group at T10H (5.2 ± 1.2 in CDR, p < 0.01 vs UT and 9.2 ± 4.8 in CR, adj-p < 0.05 vs UT).

Altogether, preconditioning the donor with CS strongly reduced the presence of CD3^pos^ T cells originating from both the donor and the recipient, during the 10-hour perfusion in the lung.

### Preconditioning the donor with CS induces an anti-inflammatory profile in the pig alveolar lung macrophages

**Figure 5:**
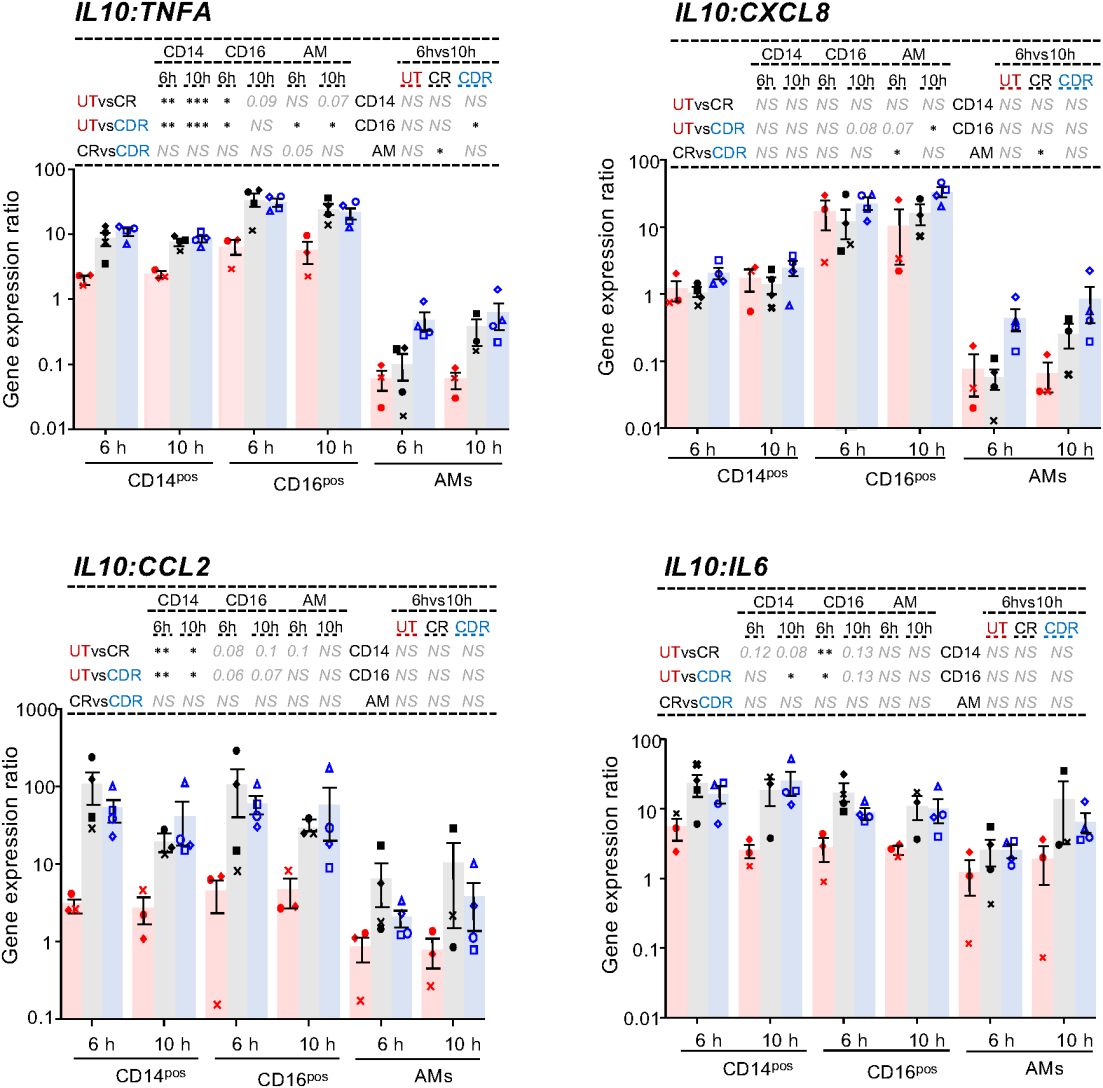
Cytokine gene expression ratios in the recipient CD14^pos^ and CD16^pos^ CFSE^pos^ MoCs and in AMs upon cross-circulation and effects of CS treatment. Gene expression arbitrary values were calculated from RT-qPCR data normalized to a house keeping gene (RSP24) and to an internal calibrator, using the 2^−ΔΔ^CT method; the gene expression data were obtained from flow cytometry sorted CFSE^pos^CD172A^pos^CD14^pos^ cells, CFSE^pos^CD172A^pos^CD16^pos^ cells and AMs from the UT (3 pigs, red), CR (4 pigs, black) and CDR groups (4 pigs, blue). A ratio between the IL10 gene expression values and the different inflammatory cytokine values was calculated. The RT-qPCR 2^−ΔΔ^CT of TNFA, IL10, CXCL8, CCL2, IL6 results are shown in **Supplementary File 12**. As the log-transformed values passed the normality test, a paired t-test was used to identify statistically significant differences between timings and an unpaired t-test was used to identify statistically significant differences between groups. The p-values were corrected for multiple testing, * < 0.05, ** < 0.01, *** < 0.001, NS stands for non-significant, p-values > 0.05 and < 0.2 are indicated.

In murine LT models, recipient classical MoCs and the donor AMs have been shown to play important roles in driving the primary graft dysfunction (2). We thus examined the effect of donor preconditioning on the activation and anti-inflammatory profile of recipient MoCs and donor AMs, in our pig model. For the activation profile analysis, the whole MoC and the AM populations were considered, as we were not able to distinguish the CD14^pos^ from the CD16^pos^ MoC subset, due to lack of convenient marker combinations. We found that preconditioning the donor strongly reduced the expression of MHC class II and CD80/86 on donor MoCs already at T0H, while this reduction needed 10-hour reperfusion to be reached in the CR group (**Additional File 10**). However, preconditioning the donor did not reduce the activation markers’ expression on AMs. For the anti-inflammatory profile analysis, we measured the expression of inflammatory genes in the recipient CD14^pos^ and CD16^pos^ MoC subsets and in the donor AMs, and we calculated the IL10:TNFA, IL10:CXCL8, IL10:CCL2 and IL10:IL6 expression ratios (**Figure 5**, **Additional File 11**). Interestingly, in the AMs, the IL10:TNFA and IL10:CXCL8 ratios were more clearly enhanced in the CDR than in the CR group (adj-p < 0.05 for CDR vs UT in most cases, and there was no significance between CR vs UT). The anti-inflammatory balance was similarly higher in the recruited MoC subsets of the CDR and CR groups than in the UT group.

Therefore, preconditioning the donor in addition to treating the recipient with CS promotes the anti-inflammatory balance in AMs.

### Preconditioning the donor with CS reduces the gene expression of T cell-attracting chemokines in AMs in pigs

**Figure 6:**
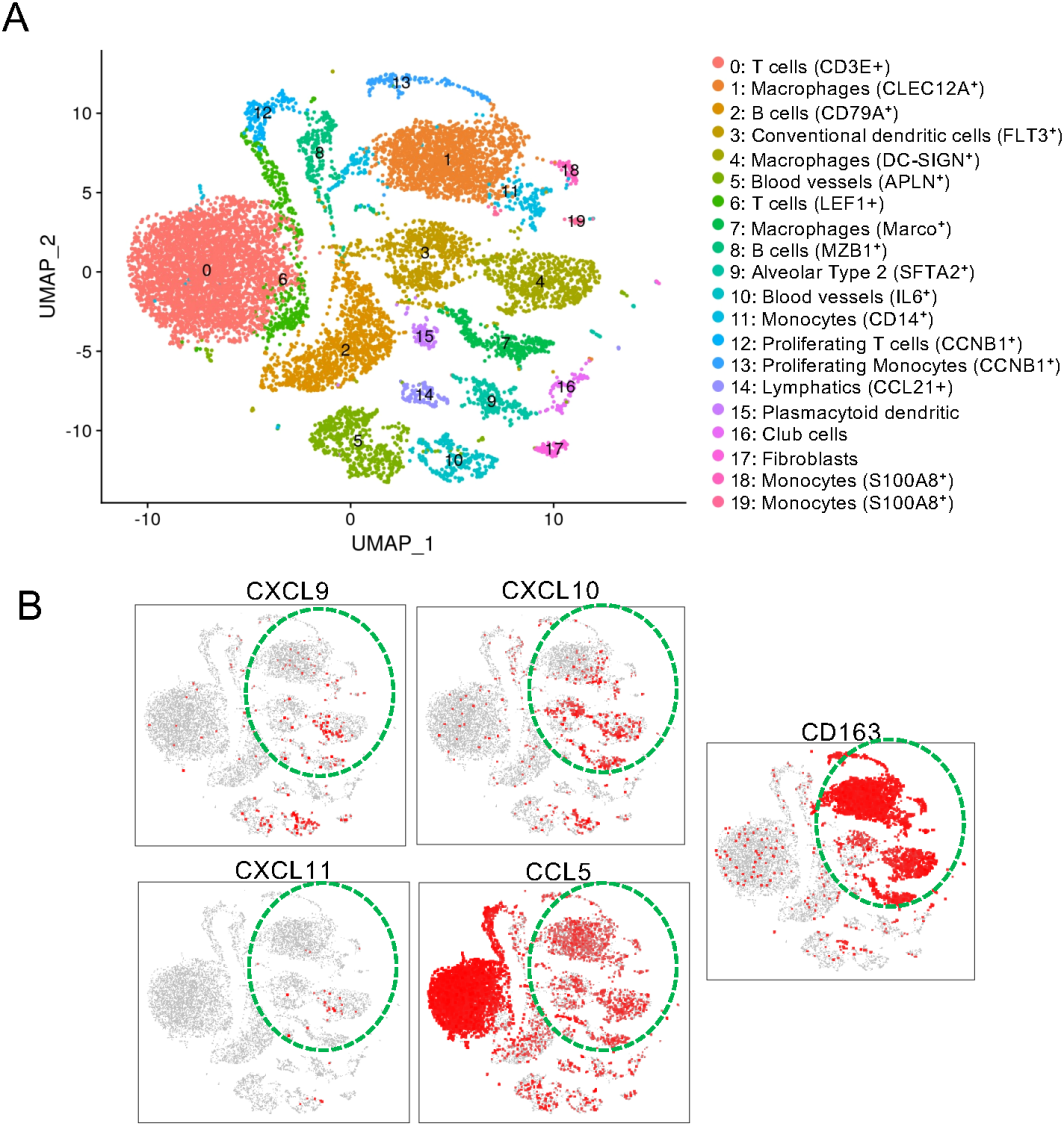
Macrophages are major expressors of CXCL9,10 and 11 transcripts in the pig lung. **(A)** Single cell RNA-seq was conducted with the 10X genomics technology (v3 chemistry) on 20 x 10^4^ isolated cells from a pig lung (post isolation with anti-MHC class II immunobeads). The data were pre-processed for a high quality transcriptome (> 500 genes and < 50000 reads per cell), clustered using the Seurat package and projected into a UMAP reduced space. The clusters were annoted based on the top marker genes extracted from each cluster using the Seurat FindMarkers function (**Additional File 5**). **(B)** The gene expressions for CD163, CCL5, CXCL9, 10 and 11 are displayed in the UMAP space with the red color representing the maximal expression level and the gray color representing absence of expression. The monocyte/macrophage cell types are indicated by a green dashed line.

**Figure 7:**
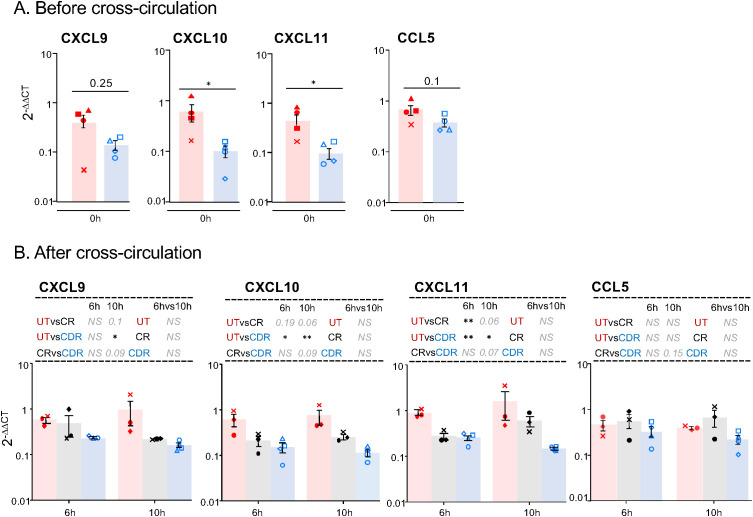
Preconditioning the donor with CS reduces the gene expression of T-cell-attracting chemokines in AMs. In **(A)** the AMs from lung biopsies collected at T0H of 4 untreated pigs and of 4 corsticosteroid treated pigs were sorted with flow cytometry. In **(B)** the AMs from lung biopsies collected after T6H and T10H cross-circulation in the UT group (3 pigs), CR group (3 pigs) and CDR group (4 pigs) were sorted with flow cytometry. In **(A, B)** the CFSE^neg^CD163^hi^ CD172A^hi^ SSC^hi^ gating was used for the AM sorting, the RNA of the sorted cells was extracted and subjected to RT-qPCR for CXCL9, CXCL10, CXCL11 and CCL5 detection. Gene expression arbitrary values were calculated from RT-qPCR data normalized to a house keeping gene (RSP24) and to an internal calibrator, using the 2^−ΔΔ^CT method. As the log-transformed values passed the normality test, a paired t-test was used to identify statistically significant differences between timings and an unpaired t-test was used to identify statistically significant differences between groups. The p-values were corrected for multiple testing, * < 0.05, ** < 0.01, NS stands for non-significant, p-values > 0.05 and < 0.2 are indicated.

We explored putative mechanisms that may explain the CD3^pos^ T cell-decrease induced by preconditioning. We checked that cell death was not increased by the CS treatment in the CD3^pos^ T cells of the three groups (**Additional File 12**). The CXCL9/CXCL10-CXCR3 axis is a target of the CS-mediated depletion of CD4^pos^ T cells in glomerulonephritis (18). As the CXCR3-interacting chemokines (CXCL9, CXCL10 and CXCL11) are produced by human AMs (19, 20), we analyzed their transcript expression in the single cell RNA-seq (scRNA-seq) data of MHC class II^pos^ pig lung cells (**Figure 6**). The UMAP revealed 20 clusters that correspond to epithelial cells, fibroblasts, vascular cells, lymphocytes, dendritic cells, monocytes, and macrophages (**Figure 6A**, **Additional File 5**, see an interactive viewer https://tinyurl.com/yr3375sd). CXCL9, CXCL10 and CXCL11 were dominantly expressed by macrophage clusters that also express CD163, and almost not by lymphoid nor epithelial cells. CCL5, another T-cell attracting chemokine was expressed by all cell types (**Figure 6B**). These chemokine genes were also found expressed in inflammatory endothelial cells (cluster 10). We therefore sorted the CFSE^neg^CD163^hi^ cells (i.e. AMs) from our control and CS-treated groups. Compared to AMs from untreated pigs, AMs from CS pigs expressed less CXCL10 and CXCL11 mRNA at T0H, p < 0.05 (**Figure 7A**). Furthermore, upon cross-circulation, the CXCL10 and CXCL11 gene expression was significantly reduced in the AMs from the CDR group at T6H (adj-p < 0.05 vs UT) and T10H (adj-p< 0.01 vs UT), whereas it was less the case in the CR group (**Figure 7B**).

Therefore, preconditioning the pig lung donor with CS leads to a sustained decrease in T cell-attracting chemokine gene expression by AMs.

### CS reduces the expression of T-cell-attracting chemokine transcripts and enhances the anti-inflammatory profile in human lung macrophages

**Figure 8:**
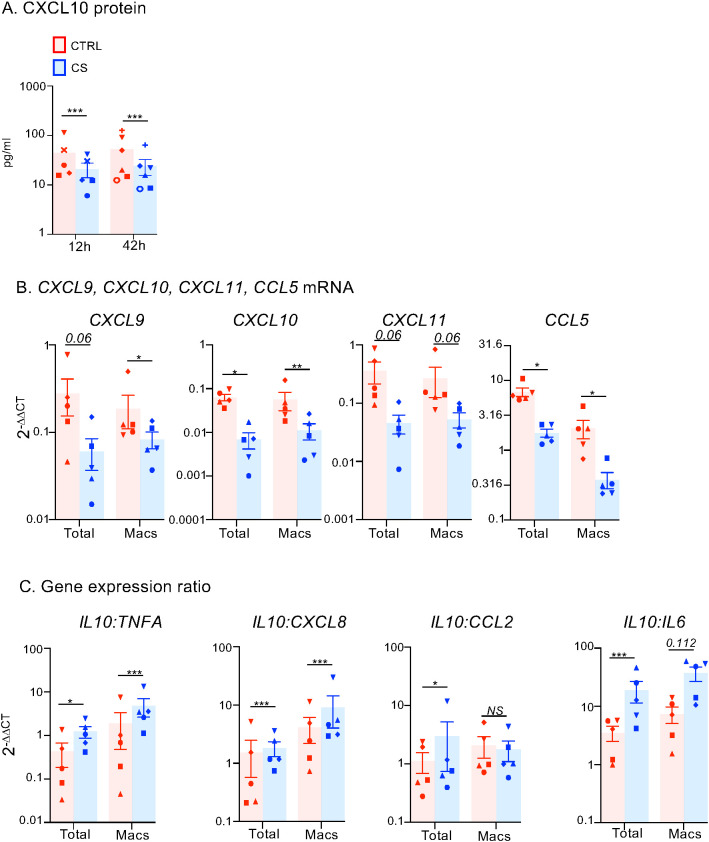
Effects of CS treatment on T-cell-attracting chemokine and inflammatory gene expression in human lung cells and macrophages. **(A)** Human lung cells from 8 patients were cultured without or with 20 µg/ml methylprednisolone (CS). The CXCL10 protein levels in the supernatants were measured by ELISA after 12 and 42 h. A specific shape symbol is attributed to each patient. **(B, C)** Human lung cells from 5 patients were cultured for 12 h without or with 20 µg/ml methylprednisolone, then detached with accutase and CD172A^pos^ macrophages were immunomagnetically sorted and then lysed for RNA extraction. Gene expression arbitrary values were calculated from RT-qPCR data normalized to a house keeping gene (RSP18) and to an internal calibrator, using the 2-DDCT method. A specific shape symbol is attributed to each patient. In B, gene expression data for T-cell attracting chemokines and CD172A are shown. In C, gene expression ratios for inflamamatory genes are shown. When the log-transformed values passed the normality test, a paired t-test was used to identify statistically significant differences (CCL5, CXCL9, CXCL10, IL10:cytokine ratios); alternatively a wilcoxson test was used (CXCL11). The p-values are reported as * < 0.05, ** < 0.01. *** < 0.005. NS, non significant.

In order to assess whether CS are also able to modify the human lung immunological profile, total human lung cells from surgical resections were cultured with and without 20 µg/ml methylprednisolone - the plasma dose obtained in humans on bolus injection. First, CS significantly reduced CXCL10 protein produced by human lung cells (**Figure 8A**, p < 0.005). As in pigs, scRNA-seq analysis showed that CXCL9, CXCL10 and CXCL11 mRNA were also mainly synthetized by lung macrophages and inflammatory endothelial cells while CCL5 transcripts were expressed by most cell types (**Additional File 13**).

After 12-hour culture of human lung cells and cell detachment with accutase, we immunomagnetically-sorted the human macrophages based on CD172A expression. We did not use the CD163 marker as it is accutase-sensitive (21). The CS-treated human lung macrophages expressed significantly less CCL5, CXCL9, 10 and 11 transcripts compared to controls, in agreement with the *in vivo* pig results (**Figure 8B**). Furthermore, CS promoted an anti-inflammatory profile, with higher *IL10:TNF, IL10:CXCL18* and *IL10:IL6* transcript ratios in CS-treated than in control lung macrophages (**Figure 8C**). As in pigs, the *IL10:CCL2* ratio was not significantly modified. These *in vitro* results suggest that the reduction of the T-cell attractive chemokine gene expression and the higher anti-inflammatory profile induced by preconditioning the donor in the pig model is likely to be translatable to humans.

Altogether our findings in a pig cross-circulation model and *in vitro* with human lung cells support that preconditioning the donor with CS improves the lung immunological environment.

## Discussion

Our study shows that donor preconditioning combined with recipient treatment with CS induced broader immunomodulation than recipient treatment alone, particularly by targeting monocytes/macrophages, which are key cell types driving primary graft dysfunction and rejection (1, 2). Indeed, a pulse of methylprednisolone given to both donor and recipient pigs reduced the T-cell attracting chemokine gene expression and promoted a more pronounced anti-inflammatory gene profile in AMs, from lung procurement through at least 10 hours post-reperfusion. This AM response was linked to a marked reduction of donor- and recipient-derived T cells in the graft. Furthermore, CS similarly modulated gene expression in human lung macrophages, underscoring translational relevance.

The treatment of deceased human donors with CS has been shown to reduce CXCL10 mRNA expression in liver grafts and, in parallel, to decrease the incidence of acute rejection in recipients (22), suggesting that preconditioning the donor with CS and dampening CXCL10 may have an impact on transplantation outcome. The mechanism by which CS regulates CXCL10 expression may vary by organ. Indeed, in the kidney, epithelial cells are the primary source of CXCL10 and prednisolone reduces its expression, thereby lowering CD4^pos^ T-cell kidney infiltration in the context of glomerulonephritis (18). In contrast, in both pig and human lungs, we found that macrophages are the main producers of CXCL10, and corticosteroids reduce its expression in these cells. Furthermore, CS reduced both CD4^+^ and CD8^+^ T-cell presence in lungs, whereas in the kidneys, only CD4^+^ T-cell levels were affected.

Donor preconditioning with CS also increased the representation of CD14^pos^ MoCs and granulocytes among the recruited (CFSE^pos^) cells in the graft, without affecting the overall immune cell recruitment (**Additional File 8**). This myeloid shift may simply reflect the reduced representation of T cells and NK cells. However, CS are also known to alter the mobilization of myeloid cells through modifications of adhesion molecule expression and responses to chemokines. For instance, CS have been shown to induce PMN mobilization by reducing their CD62L expression and upregulating the tissue expression of CXCL1, CXCL5, IFNγ, IL-6 and LTB4 (23). Moreover, the mobilization of monocytes by dexamethasone has been explained by increased chemotaxis toward C5a (24), as well as by enhanced expression of CXCR4 and of CCR2 on monocytes (25, 26).

How can our immunological finding related to preconditioning the donor impact the clinical outcome? The Toronto team recommends the use of 15 mg/kg methylprednisolone in the donor, because CS may improve the lung function by blunting the inflammatory response induced by brain death (5). In addition to this effect, our results show that preconditioning reduces the CD4^pos^ and CD8^pos^ T cell content in the graft, originating from both the recipient and the donor. Reducing the recipient recruitment is expected to reduce the initial T-cell allogeneic response in the graft. Indeed, a large fraction of the host T-cells (about 10% (27)), can be activated directly in the graft by donor-specific MHC molecules expressed as intact complexes on the donor antigen presenting cell (APC) surface (direct pathway) or by recipient APCs cross-dressed with donor MHC molecules (semidirect pathway) (28). Preconditioning the donor would thus reduce the initial polyclonal allo-response, and consequently lower the risk of acute rejection. On the other hand, reducing the donor-derived T-cells is expected to decrease the early development of anti-donor antibodies. A recent study reports that these antibodies arise due to donor passenger CD4^pos^ T cells providing help to the recipient B cells, through recognition of intact recipient MHC molecules (29). This transient, so called inverted direct pathway, is responsible for the early onset of anti-donor antibody production often observed in LT, which can negatively affect graft survival.

Our findings offer mechanistic insight into CS-based preconditioning, underscoring its immunomodulatory effects that persist for at least 10 hours, as well as its cross-species relevance. Nevertheless, we acknowledge several limitations. First, the cross-circulation platform used in this study, while permitting convenient and effective longitudinal immunological analyses distinguishing donor and recipient cells, serves as a surrogate for actual lung transplantation. Consequently, we aim to validate our results on CD3-T cell content and macrophage response in a full transplantation model, using a more accessible laboratory species such as the rat, which offers advantages over the pig in terms of cost and resource requirements. A second limitation resides in the use of lung lobectomy samples, as immune cells from cancer patients, particularly macrophages, can be dysregulated at the progenitor levels (30). Moreover, since lung cancer patients are frequently cigarette smokers, an additional concern is the well-documented association between smoking and corticosteroid resistance (31). Despite these factors, we found that lung macrophages isolated from lung biopsies taken at sites distant from the tumor exhibited corticosteroid sensitivity, as evidenced by reduced expression of CXCL9, CXCL10, and CXCL11. These findings are consistent with the *in vivo* results obtained in our porcine model. Nonetheless, confirmatory experiments using tissue from healthy donor lungs would be valuable to further validate these observations. Thirdly, we acknowledge that the clinical outcomes – both short or long term- remain to be investigated, along with key variables such as the optimal preconditioning window, i.e. the interval between CS administration and procurement. Exploration of alternative immunomodulatory agents is also warranted; for example, donor preconditioning with cyclosporine A has shown encouraging results in kidney transplantation (32). Finally, comparing the immunological effects of *in vivo* donor treatment with *ex vivo* preconditioning using normothermic lung perfusion may offer further insights into refining donor management strategies (33). Indeed administration of immunomodulatory treatments using *ex vivo* lung perfusion would be attractive for centers hesitant to apply systemic donor therapy.

## Conclusion

Importantly, our results showing improved early immune status in the lung support the broader adoption of corticosteroid-based donor preconditioning as part of standard care. While differences in clinical outcomes between centers that use CS-based preconditioning and those that do not remain unreported -likely due to confounding factors- our findings advocate for the implementation of prospective, controlled trials. Such studies are essential to determine whether CS preconditioning can improve post-transplant outcomes beyond modulating innate immune responses.

## Data Availability

The RNA sequencing data supporting the conclusions of this article (.fastq files) are available in the repository from the Gene Expression Omnibus under the accession number https://www.ncbi.nlm.nih.gov/geo/query/acc.cgi?acc=GSE296006. The datasets supporting the conclusions of this article are included within the article and its additional files. Further information and requests for resources and reagents should be directed to and will be fulfilled by the corresponding authors.
