# Supplementary material for: Preconditioning donors with corticosteroids improves early lung graft immunity
Source: Front Immunol. 2025 Oct 28;16:1668591. doi: 10.3389/fimmu.2025.1668591 (PMC12602223; doi:10.3389/fimmu.2025.1668591)
Supplement: Supplementary file 1 [file Presentation1.zip › Additonal file 4.DOCX]

**Additional 4. Primers for the pig and human experiments.**

| **Gene** | **Sequences or ID** |
| --- | --- |
| **Pig primers** | |
| RPS24 | F: AAGGAACGCAAGAACAGAATGAA  R:TTTGCCAGCACCAACGTTG |
| IL10 | F: GAGCCAACTGCAGCTTCCA  R: TCAGGACAAATAGCCCACTAGCTT |
| TNFA | F: TGGTGGTGCCGACAGATG  R:CAGCCTTGGCCCCTGAA |
| IL1B | F: GCCCTGTACCCCAACTGGTA  R: CCCAGGAAGACGGGCTTT |
| IL6 | F:CTGCTTCTGGTGATGGCTACTG  R:GGCATCACCTTTGGCATCTT |
| CXCL8 | F: TCCTGCTTTCTGCAGCTCTCT  R:GCACTGGCATCGAAGTTCTG |
| CXCL9 | F :CCCCAAGCCCTTCTTGTGA  R : AGACATGTTTGATCCCCATTCTTC |
| CXCL10 | F : TTCAGCTGCAGCACCATGA  R : CAGAGTCAGAAGAATAAGGCAGAAAA |
| CXCL11 | F : ATTCAAGGCTTCCCCATGTTC  R : TTTTACTCCAGGGCCAATGC |
| CCL2 | F: ACAGAAGAGTCACCAGCAGCAA  R: GCCCGCGATGGTCTTG |
| CCL5 | F: CCTCTGCCCACAGCTACCA  R: ATGGCGAGGATGACAGCAA |
| **Human primers, predesigned KiCqStart (Sigma-Aldrich, Merck)** | |
| CXCL10 | H_CXCL10_1 |
| CXCL9 | H_CXCL11_1 |
| CXCL11 | H_CXCL9_1 |
| CCL5 | H_CCL5_1 |
| TNFA | H_TNFA_1 |
| IL8 | H_IL8_1 |
| CCL2 | H_CCL2_1 |
| IL10 | H_IL10_1 |
| RPS18 | H_RPS18_1 |

The pig primers were designed with the primer express v.2 software and validated for their efficacy (> 90%) with escalating dose of reference pig cDNA. The human primers were obtained from the predesigned primers of Sigma-Aldrich, Merck, Germany.
