# Supplementary material for: Preconditioning donors with corticosteroids improves early lung graft immunity
Source: Front Immunol. 2025 Oct 28;16:1668591. doi: 10.3389/fimmu.2025.1668591 (PMC12602223; doi:10.3389/fimmu.2025.1668591)
Supplement: Supplementary file 1 [file Presentation1.zip › Additonal file 1.DOCX]

**Additional file 1. Lobectomy patients and anamnesis, non-small cell lung cancer without induction therapy (no chemotherapy, no immunotherapy)**

| Symbol on figure | Age | Sex | Tobacco | Pulmonary lobe | Treatments (no immunotherapy) |
| --- | --- | --- | --- | --- | --- |
| Oblique cross | 64 | M | Yes | Left inferior | Metformine  Dapglifozine  Cardensiel  Clopidogrel  Liptruzet |
| Straight cross | 63 | F | Yes | Right superior | Bipreterax  Resitune  Tahor  Omeprazole |
| Empty circle | 66 | F | Yes | Right superior | Venlafaxine  candesartan |
| Triangle pointing upwards | 65 | F | Yes | Left superior | Venlafaxine  Theralite  Levothyrox  Valium  Zopiclone  Melatonine |
| Squarre | 71 | F | Yes | Left superior | Cardensiel  Crestor  Anastrozole |
| Filled circle | 61 | M | Yes | Left inferior | Irbesartan  Mirtazapine |
| Diamond | 69 | F | No | Middle | Resitune  Aldactone  Alprazolam |
| Triangle pointing downwaords | 67 | F | Yes | Left superior | 0 |
