# Supplementary material for: Preconditioning donors with corticosteroids improves early lung graft immunity
Source: Front Immunol. 2025 Oct 28;16:1668591. doi: 10.3389/fimmu.2025.1668591 (PMC12602223; doi:10.3389/fimmu.2025.1668591)
Supplement: Supplementary file 1 [file Presentation1.zip › Additonal file 2.DOCX]

**Additional file 2. AbTable. Abs (primary mAbs and secondary Abs) used in the study**

| Primary antibodies | Provider | Catalog  number | Identity^1^ | Isotype (murine) | Concentration of use (μg/ml or dilution) |
| --- | --- | --- | --- | --- | --- |
| Anti-swMHCII | WSU | PG2006 | MSA3 | IgG2a | 2 µg/ml |
| Anti-swCD172A | WSU | PG2031 | 74.22.15A | IgG2b | 2 µg/ml |
| Anti-panCD172A | BIO-RAD | MCA6079 | DH59B | IgG1 | 5 µg/ml |
| Anti-huCD80/86 | WSU | ANC-501-020 | CTLA4-muIg^1^ | IgG2a | 5 µg/ml |
| Anti-swCD16 | BIO-RAD | MCA1971GA | G7 | IgG1 | 1/100 |
| Anti-huCD14 | Thermo-Fisher | MHC400 | TUK-4 | IgG2a | 1/33 |
| Anti-swCD8α | WSU | PG2018 | PT81B | IgG2b | 2 µg/ml |
| Anti-swCD335 | BIO-RAD | MCA5972GA | VIVKM1 | IgG1 | 2 µg/ml |
| Anti-swCD4 | WSU | PG2013 | PT90A | IgG2a | 2 µg/ml |
| Anti-swCD3 | Clinisciences | 4510-01 | PPT3 | IgG1 | 2.5 µg/ml |
| Anti-swgranulocyte | WSU | PG2045 | PG68A | IgG1 | 2 µg/ml |
| Anti-huCD21 | BD-Biosciences | 555421 | B-Ly4 | IgG1 | 5 µg/ml |
| Conjugated primary  antibodies | Provider | Catalog number | Identity^1^ | Isotype  (murine) | Concentration of use (μg/ml or dil) |
| Anti-swCD163-PE | BIO-RAD | MCA2311PE | 2A10/11 | IgG1 | 1/20 |
| Anti-swCD3-PE | BIO-RAD | MCA5951PE | PPT3 | IgG1 | 1/10 |
| ISC-G1-PE | Invitrogen | [12-4714-82](https://www.thermofisher.com/antibody/product/Mouse-IgG1-kappa-clone-P3-6-2-8-1-Isotype-Control/12-4714-82) | P3.6.2.8.1 | IgG1 | 1/10 |
| Secondary Antibodies | Provider | Catalog number | Identity^1^ |  | Concentration of use (μg/ml or dil) |
| Goat anti-mu IgG2b-APC-Cy7 | Abcam | ab130791 | Goat polyclonal IgG | | 1/100 |
| Rat anti-mu  IgG1-PerCP-eFluor710-Cy5.5 | Fisher | 15361310 | Rat mAb M1-14D12 | | 1/200 |
| Goat anti-mu  IgG2a-A647 | Invitrogen | A-21241 | Goat polyclonal IgG | | 1/200 |

^1^ Identity corresponds either to the original clone (mAb), a fusion protein (CTLA4-muIg, i.e. human CTLA4 fused to murine IgG2a sequences), or a polyclonal IgG (species of origin). ^2^The species of the targeted molecule is either swine (sw), murine (mu), human (hu), or pan-species (pan for swine, bovine, human).
