# Supplementary material for: Preconditioning donors with corticosteroids improves early lung graft immunity
Source: Front Immunol. 2025 Oct 28;16:1668591. doi: 10.3389/fimmu.2025.1668591 (PMC12602223; doi:10.3389/fimmu.2025.1668591)
Supplement: Supplementary file 1 [file Presentation1.zip › Additional files 3-6-8-10-11-12-13.PDF]

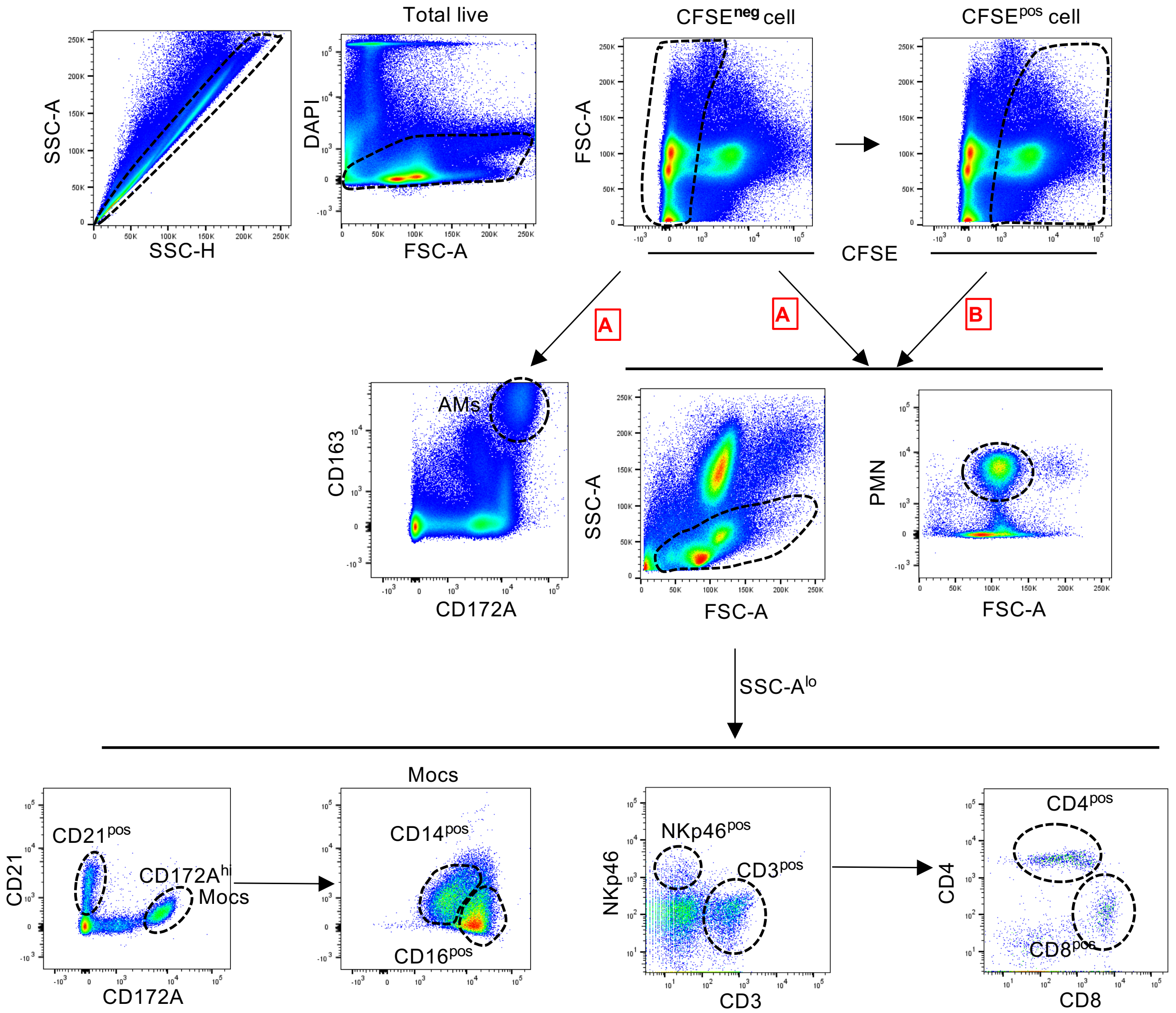


**Additional file 3. Gating strategy for the pig lung immune cell analyses, extracted from the supplementary material of our recent paper [Glorion, 2023, PMID 37253049].** After gating on singlet and live cells, the workflow A was followed for CFSE$^{neg}$ cells analyses and the workflow B was followed for CFSE$^{pos}$ cell analyses. Except for PMNs and alveolar macrophages (AMs), cells were analyzed using an intermediate gate on SSC-A$^{lo}$ cells to avoid noises from PMNs. AMs, PMNs, CD172A$^{hi}$ (monocytic cells, MoCs), CD14$^{pos}$ and CD16$^{pos}$ monocytic cells, CD21$^{pos}$ (B cells), NKp46$^{pos}$ (NK cells), CD21$^{pos}$(B cells), CD3$^{pos}$ T cells, CD4$^{pos}$ T cells, CD8$^{pos}$ T cell subsets are shown.

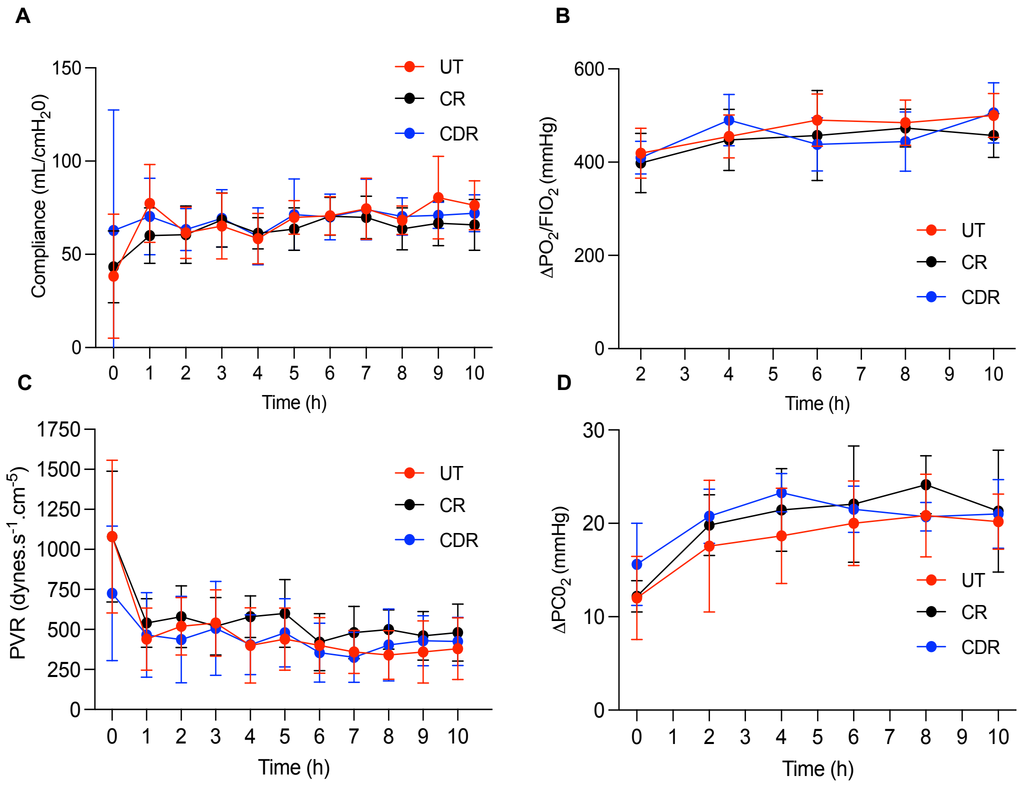


**Additional file 6. Extracorporeal lung stability and performance throughout 10 h of cross-circulation. A**. Static compliance. **B.** $\Delta PO_2/FIO_2$: venous $PO_2$ – arterial $PO_2$/ $FIO_2$. **C.** Pulmonary Vascular Resistance (PVR): (pulmonary arterial pressure - left atrium pressure) x 80/flow rate. **D.** $\Delta PCO_2$ = arterial $PCO_2$ – venous $PCO_2$ **.** Pulmonary Vascular Resistance (PVR): (pulmonary arterial pressure - left atrium pressure) x 80/flow rate. **In A, B, C, D** : all values represent mean ± standard deviation, untreated group (UT) values in red (n = 4), corticosteroid-Recipient (CR) values (n = 4) in black, corticosteroid-Donor-Recipient (CDR) values (n = 4) in blue. The values in the 3 groups were compared by a two-tailed Wilcoxson test at each timing and no significant differences were obtained.

| | 0h | 6h | 10h | | UT | CR | CDR |
|---|---|---|---|---|---|---|---|
| UTvsCR | *NS* | *NS* | *NS* | 0hvs6h | ** | ** | ** |
| UTvsCDR | *NS* | *NS* | *NS* | 0hvs10h | ** | ** | ** |
| CRvsCDR | *NS* | *NS* | *NS* | 6hvs10h | *NS* | *NS* | *NS* |

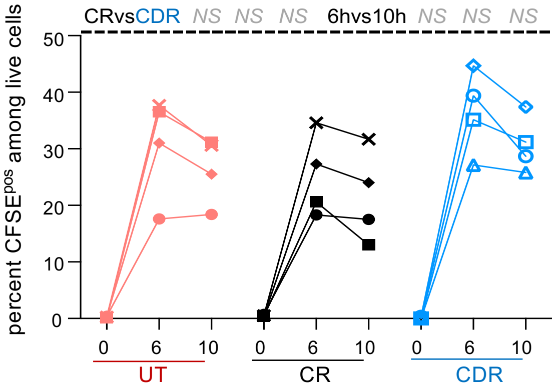


**Additional file 8. The global recruitment of recipient cells in lung graft is not affected by the corticosteroid treatmen**t. Lung biopsies were collected at 0 h, 6 h and 10 h of cross-circulation and a single cell suspension was generated by enzymatic treatment. The proportion of CFSE$^{pos}$ cells among live lung cells are shown. Each pig is labelled with a unique colored symbol throughout the paper (UT no treatment in red, CR corticosteroids to recipient only in black, CDR corticosteroids to donor and recipient in blue). As the data distribution passed the normality test, a paired t-test test was used to identify statistically significant differences between timings and an unpaired t-test was used to identify statistically significant differences between groups, with p-values reported in a table above the panel. The p-values were corrected for multiple testing, * < 0.05, ** < 0.01, *** < 0.001, NS stands for non-significant, p-values > 0.05 and < 0.2 are indicated. The mean and sd values are reported in Additional file 9.

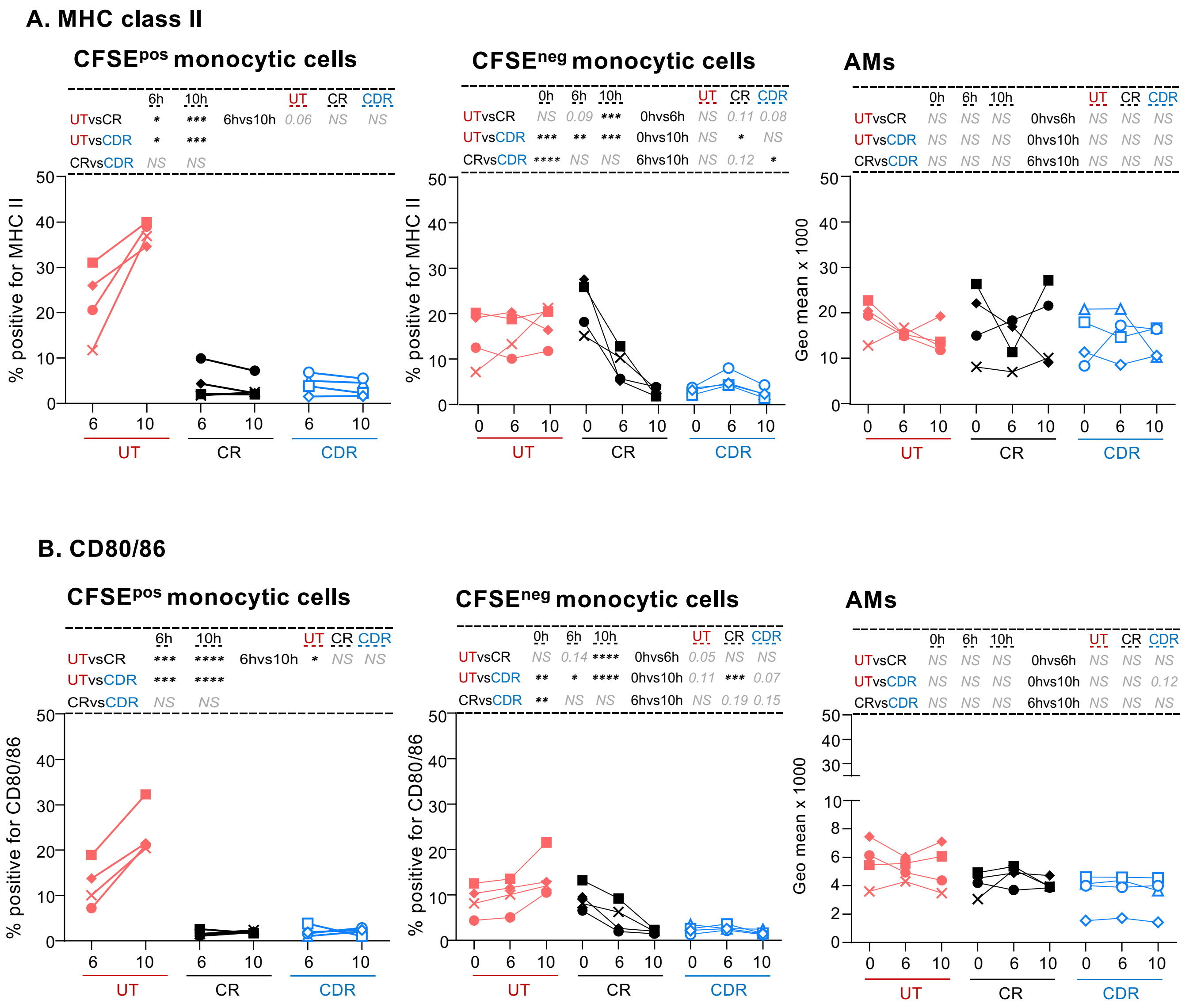


**Additional file 10. The corticosteroid treatments reduce the MHC class II and CD80/CD86 expression only on MoCs and not on AMs.** A. Percent of MHC class II$^{pos}$ cells within live CFSE$^{pos}$CD172$^{hi}$ cells (left panel), within live CFSE$^{neg}$CD172$^{hi}$ cells (middle panel) and geometric mean expression of MHC class II on AMs (that all are MHC class II$^{pos}$). B. Same as in A for the analysis of CD80/86 expression. Each pig is labelled with a unique colored symbol throughout the paper (untreated group (UT), corticosteroid-treated recipients (CR), corticosteroid-treated donors and recipients (CDR), UT in red, CR in black, CDR in blue). As log-transformed values passed a normality test, a paired t-test was used to identify statistically significant differences between timings and an unpaired t-test was used to identify statistically significant differences between the groups, with p-values reported in a table above each panel. The p-values were corrected for multiple testing, * < 0.05, ** < 0.01, *** < 0.001, NS stands for non-significant, p-values > 0.05 and < 0.2 are indicated.

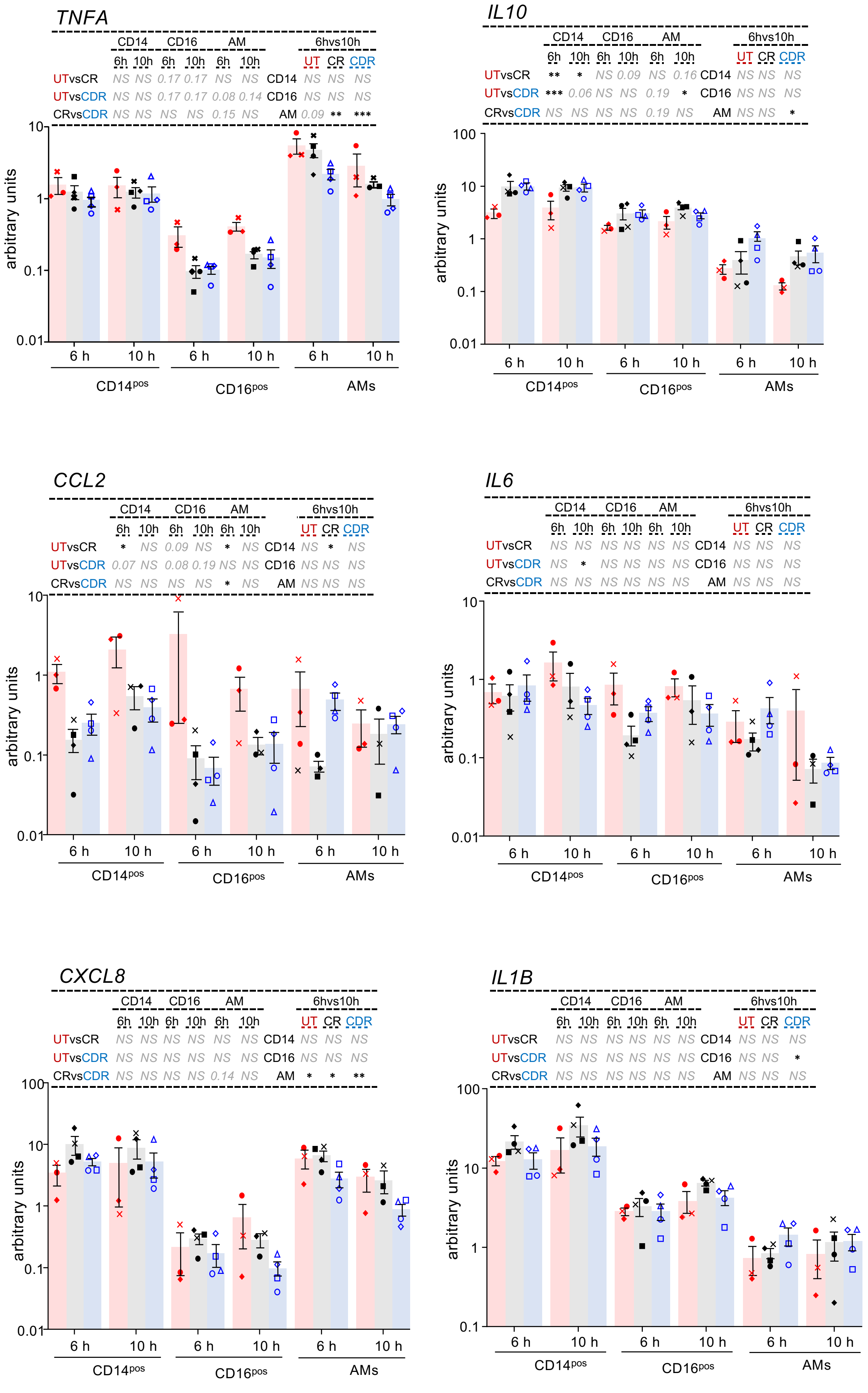


**Additional file 11. Cytokine gene expression in the CD14$^{pos}$ and CD16$^{pos}$ recruited MoCs and in AMs upon cross-circulation and effects of corticosteroid treatment**. Gene expression arbitrary values were calculated from RT-qPCR data normalized to a house keeping gene (RSP24) and to an internal calibrator, using the $2^{-\Delta\Delta}$CT method; the gene expression data were obtained from flow cytometry sorted CFSE$^{pos}$CD172A$^{pos}$CD14$^{pos}$ cells, CFSE$^{pos}$CD172A$^{pos}$CD16$^{pos}$ cells and AMs from the UT (3 pigs, red), CR (4 pigs, black) and CDR groups (4 pigs, blue). When the log-transformed values passed the normality test, a paired t-test was used to identify statistically significant differences between timings and an unpaired t-test was used to identify statistically significant differences between groups. Alternatively a wilcoxson test was used. The p-values were corrected for multiple testing, * < 0.05, ** < 0.01, *** < 0.001, NS stands for non-significant, p-values > 0.05 and < 0.2 are indicated.

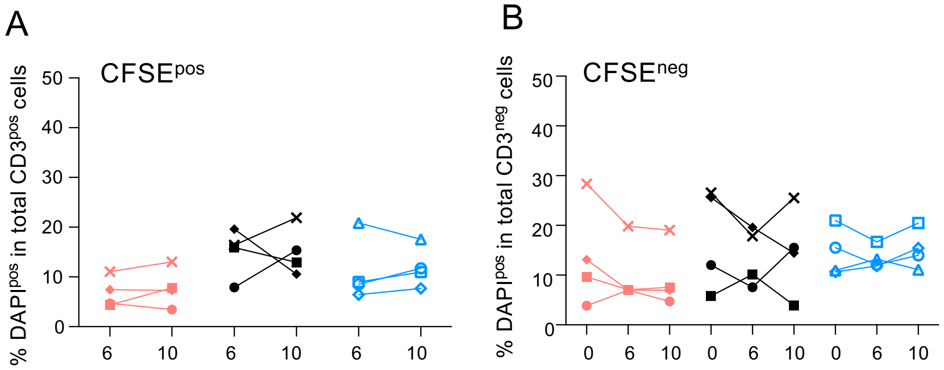


**Additional file 12. The corticoid protocols in the CR and CDR groups do not induce cell death in CD3$^{pos}$ T cells**. Isolated lung cells were gated on CFSE$^{pos}$ and CFSE$^{neg}$ cells, and the % DAPI$^{pos}$ cells was analysed among the CD3$^{pos}$ T cells. There was no statistically significant differences between groups nor timing.

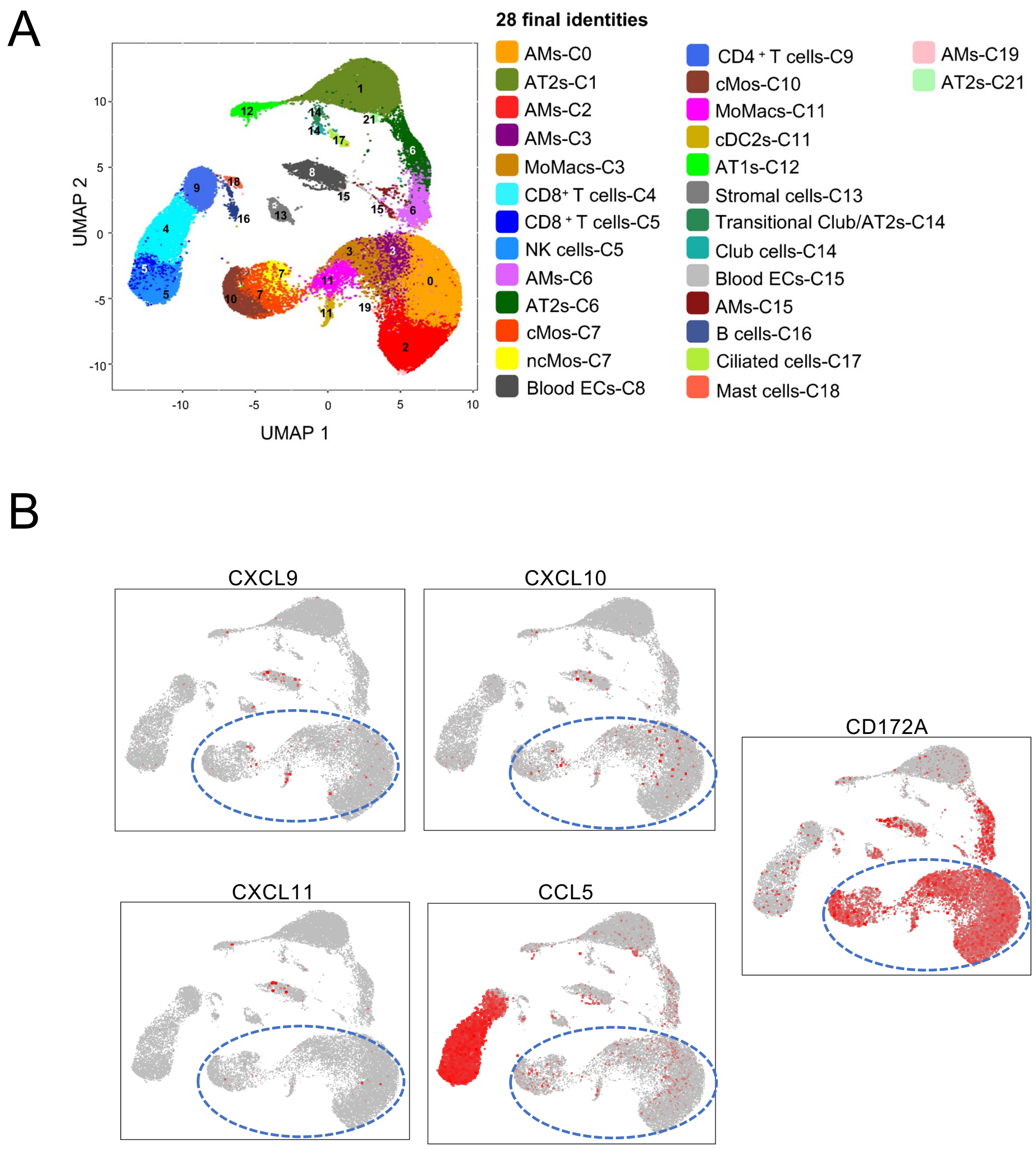


**Additional file 13. Macrophages are major expressors of CXCL9,10 and 11 transcripts in the human lung**. **A.** Single cell RNA-seq was conducted with the 10X genomics technology (v3 chemistry) on isolated human lung cells, as thoroughly described in the figure 3 of our previously published study (Vu Mahn et al, 2024, PMID 39147987). The cells originated from human lungs before and after *ex vivo* lung perfusion with or without SARS-CoV-2 exposure. The cell identity defined by Azimuth algorithm and cluster belonging are projected on the UMAP (21 clussters, 28 identities, see (Vu Mahn et al, 2024, PMID 39147987). Note that cluster 6 correspond to cell doublets including macrophages. **B.** The gene expressions for CD172A, CCL5, CXCL9, 10 and 11 are displayed in the UMAP space with the red color representing the maximal expression level and the grey color representing absence of expression. Only cells originating from non-infected lungs before perfusion were selected for generating the UMAPs in B. The monocyte/macrophage cell types are indicated by a blue dashed line.
