# Supplementary material for: Preconditioning donors with corticosteroids improves early lung graft immunity
Source: Front Immunol. 2025 Oct 28;16:1668591. doi: 10.3389/fimmu.2025.1668591 (PMC12602223; doi:10.3389/fimmu.2025.1668591)
Supplement: Supplementary file 1 [file Presentation1.zip › Additonal file 7.DOCX]

| Parameter | Group | Time (h) | | | | | | | | | | |
| --- | --- | --- | --- | --- | --- | --- | --- | --- | --- | --- | --- | --- |
|  |  | 0 | 1 | 2 | 3 | 4 | 5 | 6 | 7 | 8 | 9 | 10 |
| Heart rate  (bpm) | UT | 95±24.9 | 92±24.9 | 88.8±13.1 | 81±16.9 | 90.6±22.9 | 90.2±20.5 | 85.2±19.1 | 92.6±19.8 | 91.2±5.8 | 95±11.9 | 100.4±14.1 |
|  | CR | 113±46.6 | 106.3±45.7 | 104.5±46.7 | 107.3±45.1 | 85±11.6 | 86.5±7.4 | 90±6.3 | 84±8.1 | 81.5±15.9 | 82.3±15.7 | 96.3±12.8 |
|  | CDR | 104.5±7.5 | 98.8±7.8 | 89.0±12.5 | 81.0±2.4 | 87.5±13.6 | 88.5±18.4 | 81.3±2.2 | 87.3±18.3 | 92.5±16.5 | 88.7±16.0 | 89.8±20.9 |
| Systolic BP  (mm hg) | UT | 89±9.8 | 97.8±9.1 | 94±10.7 | 89.6±11.03 | 88.8±9.9 | 93.8±3.9 | 81.8±12.1 | 96±4.7 | 100±6.6 | 97.8±8.1 | 92±3.1 |
|  | CR | 98.3±20,0 | 96.5±6.7 | 103.8±2.6 | 92.0±14.2 | 91.0±9.7 | 89.5±9.8 | 94.0±13.7 | 91.3±11.3 | 90.0±13.3 | 90.0±13.3 | 81.8±6.7 |
|  | CDR | 106.7±11.1 | 101.8±4.3 | 94.0±13.0 | 91.0±10.2 | 97.8±12.1 | 84.3±6.2 | 85.0±11.2 | 85.0±9.3 | 83.0±14.1 | 86.0±14.0 | 85.0±13.5 |
| Temperature  (°c) | UT | 37.6±1.1 | 37.9±0.8 | 38.3±0.6 | 38.5±0.6 | 38.8±0.6 | 38.5±0.2 | 38.5±0.3 | 38.45±0.3 | 38.5±0.3 | 38.5±0.3 | 38.5±0.4 |
|  | CR | 37.2±0.5 | 38.24±0.5 | 38.5±0.6 | 38.6±0.5 | 38.7±0.3 | 38.6±0.4 | 38.5±0.3 | 38.5±0.2 | 38.5±0.1 | 38.5±0.1 | 38.5±0.1 |
|  | CDR | 37.6±1.1 | 37.9±0.8 | 38.3±0.6 | 38.5±0.6 | 38.8±0.6 | 38.5±0.2 | 38.5±0.3 | 38.45±0.3 | 38.5±0.3 | 38.5±0.3 | 38.5±0.4 |
| PH | UT | 7.3±0.04 |  | 7.4±0.03 |  | 7.5±0.04 |  | 7.5±0.04 |  | 7.4±0.03 |  | 7.4±0.3 |
|  | CR | 7.3±0.01 |  | 7.4±0.05 |  | 7.4±0.04 |  | 7.4±0.05 |  | 7.4±0.06 |  | 7.4±0.04 |
|  | CDR | 7.3±0.06 |  | 7.4±0.02 |  | 7.4±0.02 |  | 7.4±0.03 |  | 7.4±0.01 |  | 7.4±0.03 |
| Lactate  (mmol/l) | UT | 4.9±3.3 |  | 1.5±0.8 |  | 1.1±0.7 |  | 1.0±0.4 |  | 0.9±0.5 |  | 0.8±0.2 |
|  | CR | 3.2±0.8 |  | 2.1±1.4 |  | 1.4±0.6 |  | 1.0±0.2 |  | 0.9±0.4 |  | 0.7±0.1 |
|  | CDR | 5.7±3.6 |  | 1.3±0.9 |  | 1.1±0.2 |  | 0.9±0.2 |  | 0.8±0.1 |  | 0.8±0.3 |
| Creatinine  (mmol/l) | UT | 119.2±25.3 |  | 120±1.4 |  | 127.2±14.4 |  | 137.3±17.5 |  | 148.3±24.9 |  | 150.4±26.9 |
|  | CR | 101.8±2.5 |  | 131.3±12.7 |  | 131.5±23.6 |  | 131.6±33.4 |  | 140.5±32.8 |  | 145.6±43.1 |
|  | CDR | 85.0±20.7 |  | 99.0±13.1 |  | 105.0±21.5 |  | 112.7±18.5 |  | 129.5±13.7 |  | 135.3±18.6 |
| Haemoglobin  (g/dl) | UT | 8.9±1.6 |  | 6.7±0.4 |  | 6.9±0.2 |  | 7.3±1.5 |  | 8.2±1.5 |  | 6.8±0.6 |
|  | CR | 9.2±0.6 |  | 7.9±2.0 |  | 8.6±2.7 |  | 9.2±3.3 |  | 8.3±2.3 |  | 8.5±2.9 |
|  | CDR | 7.4±2.0 |  | 8.35±1.2 |  | 7.0±0.6 |  | 8.2±2.5 |  | 8.2±5.1 |  | 6.9±2.4 |
| Glucose  (mmol/l) | UT | 7.1±2.2 |  | 5.8±0.3 |  | 6.2±1.2 |  | 6.7±1.3 |  | 6.6±1.7 |  | 5.9±1.6 |
|  | CR | 6.15±0.7 |  | 6.7±1.3 |  | 5.4±3.2 |  | 7.5±2.1 |  | 5.9±1.2 |  | 7.3±2.4 |
|  | CDR | 4.7±2.2 |  | 7.9±0.6 |  | 8.7±1.1 |  | 6.7±3.4 |  | 7.8±0.8 |  | 6.1±3.0 |
| Activated clotting time (s) | UT | 167.8±32.5 |  | 171±16.5 |  | 177.4±10.8 |  | 166.6±18.8 |  | 156.4±25.5 |  | 174.3±15.6 |
|  | CR | 190.8±25.7 |  | 189.8±16.5 |  | 173.2±15.7 |  | 180.8±8.3 |  | 181.8±21.4 |  | 185.2±21.3 |
|  | CDR | 180.5±29.1 |  | 161.8±20.9 |  | 172.3±33.0 |  | 176.8±19.4 |  | 181.3±12.9 |  | 182.8±10.7 |
| White Blood Cells (10^3^/mm^3^) | UT | 18.2±2.8 | 12.9±2.4 |  | 14.3±2.3 |  |  | 14.3±2.3 |  |  |  | 16.8±5.8 |
|  | CR | 17.6±5.2 | 14.3±2.4 |  | 18.6±4 |  |  | 18.7±4 |  |  |  | 22.5±2.9 |
|  | CDR | 15.9±4.9 | 15.8±2.2 |  | 14.6±2.1 |  |  | 22.0±8.2 |  |  |  | 18.5±1.3 |
| Lymphocytes (10^3^/mm^3^) | UT | 9.6±1.4 | 7.3±1.9 |  | 6.5±1.2 |  |  | 4.8±0.6 |  |  |  | 5.3±0.5 |
|  | CR | 10.6±2.7 | 7.1±1.3 |  | 5.1±0.6 |  |  | 4.9±1.1 |  |  |  | 5.4±0.9 |
|  | CDR | 7.4±2.1 | 5.5±1.3 |  | 4.1±1.0 |  |  | 5.26±1.6 |  |  |  | 5.3±1.1 |
| Neutrophils (10^3^/mm^3^) | UT | 7.6±2.9 | 4.3±0.8 |  | 7±2.8 |  |  | 9.9±6 |  |  |  | 10.1±5 |
|  | CR | 5.9±2.3 | 6.3±1.7 |  | 12.6±3.6 |  |  | 15.4±3.6 |  |  |  | 15.6±1.8 |
|  | CDR | 7.7±2.6 | 9.1±0.9 |  | 9.6±1.3 |  |  | 15.5±6.2 |  |  |  | 11.8±1.3 |
| Eosinophils (10^3^/mm^3^) | UT | 0.19±0.1 | 0.11±0.04 |  | 0.11±0.04 |  |  | 0.07±0.04 |  |  |  | 0.10±0.05 |
|  | CR | 0.3±0.3 | 0.5±0.8 |  | 0.14±0.07 |  |  | 0.09±0.03 |  |  |  | 0.09±0.05 |
|  | CDR | 0.1±0.05 | 0.06±0.02 |  | 0.09±0.02 |  |  | 0.08±0.08 |  |  |  | 0.7±0.05 |
| Basophils (10^3^/mm^3^) | UT | 0.09±0.09 | 0.04±0.02 |  | 0.08±0.04 |  |  | 0.11±0.05 |  |  |  | 0.05±0.07 |
|  | CR | 0.04±0.02 | 0.07±0.07 |  | 0.08±0.05 |  |  | 0.06±0.04 |  |  |  | 0.06±0.04 |
|  | CDR | 0.06±0.05 | 0.12±0.03 |  | 0.8±0.06 |  |  | 0.10±0.12 |  |  |  | 0.13±0.03 |
| Monocytes (10^3^/mm^3^) | UT | 0.76±0.09 | 0.54±0.12 |  | 0.75±0.12 |  |  | 0.6±0.07 |  |  |  | 1.2±0.6 |
|  | CR | 0.78±0.16 | 0.55±0.09 |  | 0.85±0.08 |  |  | 0.87±0.67 |  |  |  | 1.4±0.47 |
|  | CDR | 0.75±0.32 | 0.92±0.33 |  | 0.70±0.34 |  |  | 1.11±0.46 |  |  |  | 1.3±0.35 |
| Red Blood Cells (10^6^/mm^3^) | UT | 6.6±0.6 | 4.5±0.6 |  | 4.4±0.4 |  |  | 4.3±0.6 |  |  |  | 4.2±0.6 |
|  | CR | 6.1±0.5 | 5.6±0.7 |  | 5.3±0.7 |  |  | 5.1±0.5 |  |  |  | 5.0±0.6 |
|  | CDR | 8.0±2.0 | 5.1±0.6 |  | 4.7±0.6 |  |  | 5.4±1.0 |  |  |  | 4.2±0.5 |
| Platelets (10^3^/mm^3^) | UT | 323±49 | 237±90 |  | 209±70 |  |  | 204±88 |  |  |  | 190±60 |
|  | CR | 304±64 | 259±57 |  | 231±56 |  |  | 224±75 |  |  |  | 210±45 |
|  | CDR | 304±93 | 181±106 |  | 218±17 |  |  | 266±71 |  |  |  | 185±19 |
| Hematocrit  (%) | UT | 35.3±5.5 | 23.8±4.4 |  | 22.8±2.4 |  |  | 22.1±2.7 |  |  |  | 22±3.1 |
|  | CR | 32.9±3.5 | 30.1±4.1 |  | 28.2±4.1 |  |  | 26.8±3.8 |  |  |  | 26.1±3.8 |
|  | CDR | 31.3±11.0 | 25.8±2.5 |  | 24.9±2.2 |  |  | 28.4±8.2 |  |  |  | 21.7±1.5 |

**Additional file 7A: Vital and biological parameters in recipient pigs throughout 10 h of cross-circulation support.** The monitored parameters are reported for UT (red), CR (black) and CDR (blue) pig groups. Values represent mean ± standard deviation. For each value both group was compared by anova two-way for repeated measure and no differences was shown excepted for neutrophils which was higher in CR than in UT p=0.0362.

BP, blood pressure

| Pig number (UT, CR and CDR groups | Warm ischemia (min) | Cold Storage (min) |
| --- | --- | --- |
| UT1 | 84 | 95 |
| UT2 | 88 | 105 |
| UT3 | 81 | 115 |
| UT4 | 92 | 81 |
| UT Mean ± sd | 86.2 ± 4.8 | 99 ± 14.5 |
| CR1 | 87 | 98 |
| CR2 | 77 | 107 |
| CR3 | 87 | 95 |
| CR4 | 60 | 140 |
| CR Mean ± sd | 77.7 ± 12.7 | 110 ± 20.6 |
| CDR1 | 78 | 100 |
| CDR2 | 95 | 75 |
| CDR3 | 73 | 90 |
| CDR4 | 75 | 90 |
| CDR Mean ± sd | 80.3 ± 10.1 | 88.8 ±10.3 |

**Additional file 7B. Ischemic durations (warm and cold) of the donor lungs in the UT, CR and CDR groups, showing individual values, and mean ± sd.** As the data did not pass the normality test, a bilateral Mann Whitney test was performed to compare the UT, CR and CDR values and revealed no statistically significant differences.
